# Ultrasound‐based haemodynamic force trajectories in a murine model of reversible pressure overload

**DOI:** 10.1113/JP290877

**Published:** 2026-07-23

**Authors:** Peyton M. Day, Thomas Moore‐Morris, Craig J. Goergen, Pierre Sicard

**Affiliations:** ^1^ Weldon School of Biomedical Engineering Purdue University West Lafayette Indiana USA; ^2^ Université de Montpellier, INSERM, CNRS, IGF Montpellier France; ^3^ Université de Montpellier, INSERM, CNRS, PhyMedExp Montpellier France

**Keywords:** haemodynamic forces, heart failure, pressure overload, strain, unloading

## Abstract

**Abstract:**

Haemodynamic forces (HDF) quantify intraventricular pressure gradients as integrated vectors derived from myocardial motion and blood flow, providing a sensitive marker of left ventricular (LV) remodelling beyond conventional metrics such as ejection fraction or strain. Although HDF analysis has been clinically validated in heart failure, it remains underexplored in preclinical models. To determine whether HDF metrics derived from high‐resolution murine echocardiography can sensitively track cardiac dysfunction and predict functional recovery, male mice (*n* = 8) underwent serial echocardiography at baseline, after transverse aortic constriction (TAC) and 4 weeks after aortic debanding (deTAC). LV function and interval‐specific HDF components were quantified, including longitudinal and transverse forces over predefined systolic and diastolic windows. Longitudinal interval‐based parameters emerged as sensitive, integrative markers of LV dysfunction after TAC and deTAC. Notably, a novel exploratory waveform, early‐to‐late systolic impulse longitudinal HDF ratio, showed the strongest associations with pressure overload and subsequent functional recovery (baseline *vs*. TAC: *P* < 0.001; TAC *vs*. deTAC: *P* = 0.003; baseline *vs*. deTAC: *P* = 0.049). By contrast, diastolic HDF indices, including the previously proposed longitudinal e‑wave ratio, did not significantly differentiate between time points. Transverse HDF over the systolic impulse interval further demonstrated potential for predicting post‑surgical recovery following unloading. These findings support systolic HDF metrics, particularly longitudinal and transverse interval‑based parameters, as sensitive integrative markers of LV dysfunction and reverse remodelling in preclinical pressure‑overload models, and highlight their translational relevance for echocardiography‑based HDF analysis.

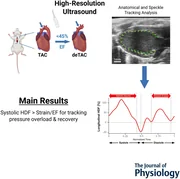

**Key points:**

- Haemodynamic force (HDF) analyses evaluate intraventricular pressure gradients as integrated vectors of myocardial motion and blood flow, providing complementary markers of pump (in)efficiency beyond ejection fraction or strain.
- High‐resolution murine echocardiography enables robust derivation of interval‐specific HDF metrics.
- Systolic HDF metrics, particularly transverse components during impulse intervals, showed potential for predictive power correlating with cardiac recovery.
- Systolic HDF reflect key changes in force generation and intraventricular flow during pressure overload, allowing early detection of maladaptive remodelling and treatment response, with translational potential for non‐invasive risk stratification in aortic stenosis patients.

## Graphical abstract

**Abstract figure legend** High‐resolution ultrasound anatomical imaging and speckle‐tracking analysis were used to compute left ventricular (LV) haemodynamic forces (HDF) in a murine model of transverse aortic constriction (TAC) followed by aortic debanding (deTAC). Representative curves show typical LV longitudinal components of haemodynamic force plotted over a single, time‐normalized cardiac cycle. EF, ejection fraction.

## Introduction

Cardiovascular diseases including heart failure are one of the leading causes of death among people in the developed world, resulting in 19.2 million global deaths in 2023 and impacting 64 million people a year (Global Burden of Cardiovascular Diseases & Risks 2023 Collaborators, 2025). Despite major therapeutic advances, clinical outcomes remain highly dependent on the ability to accurately monitor disease progression and cardiac recovery, underscoring the need for sensitive and mechanistically meaningful biomarkers of cardiac function (Aimo et al., 2022). Conventional imaging metrics, including ventricular volumes, ejection fraction, and myocardial strain, primarily describe myocardial deformation but provide limited insight into the intracavitary forces that govern blood transport. Because ventricular filling and ejection are fundamentally driven by intraventricular pressure gradients (IVPGs), metrics that capture the interaction between myocardial motion and intracardiac fluid dynamics may offer complementary and potentially earlier indicators of functional impairment and recovery (Sørensen et al., 2025). In this context, haemodynamic forces (HDF) have emerged as quantitative descriptors of cardiac performance by estimating the pressure forces responsible for blood acceleration within the heart (Vallelonga et al., 2021). HDF reflect the dynamic balance between blood inertia and pressure fields generated by myocardial contraction and relaxation, thereby providing a direct mechanical link between myocardial deformation, chamber geometry and intracardiac flow dynamics (Greenberg et al., 2001). Initially introduced more than two decades ago using Doppler M‐mode echocardiography (Greenberg et al., 2001), HDF analysis was subsequently validated using four‐dimensional (4D) flow magnetic resonance imaging (MRI) (Pedrizzetti et al., 2014, 2017) and has demonstrated clinical relevance in several pathological settings. Notably, recent studies have shown that HDF metrics can predict long‐term responses to cardiac resynchronization therapy in patients with left bundle branch block (Eriksson et al., 2017; Laenens et al., 2024), highlighting their potential as clinically actionable biomarkers of ventricular mechanics. Despite these promising clinical results, HDF metrics remain largely underexplored in preclinical research, particularly in small animal studies. Although, one study employed MRI‐based approaches for HDF assessment (Daal et al., 2022), no investigations to date have leveraged high‐resolution echocardiography in rodent models, an advancement that could enable cost‐effective longitudinal studies with greater accessibility and earlier detection of pathologies. The present study is thus positioned to expand the findings of human research using ultrasound (Cesareo et al., 2024; Dal Ferro et al., 2019; Faganello et al., 2024; Pedrizzetti et al., 2016, 2017; Vairo et al., 2023) to a murine model that has been previously investigated using cardiovascular magnetic resonance (Daal et al., 2022).

In the present study, we aimed to determine whether high‐resolution ultrasound‐derived HDF serves as a sensitive, mechanistically meaningful marker of left ventricular (LV) adaptation and recovery in the murine transverse aortic constriction (TAC)/aortic debanding (deTAC) model, a surgical protocol of aortic constriction and subsequent debanding. The TAC/deTAC model enables the evaluation of dynamic changes in ventricular mechanics during loss and recovery of LV function (Ghajar‐Rahimi et al., 2026). Given the inherent complexity of HDF waveforms and their derived parameters based on intraventricular pressure gradients, a central aim of this work was to identify HDF metrics that are most representative of cardiac functional status and to establish robust quantification strategies for their extraction in a preclinical ultrasound setting. Specifically, we tested whether interval‐specific systolic HDF metrics capture changes in intraventricular force generation and flow turbulence induced by TAC, and whether these parameters may better predict functional recovery after deTAC than conventional echocardiographic indices.

## Methods

### Ethical approval

Experiments were conducted following European Directive (2010/63/EU) and French laws for laboratory animal use. The local institutional ethics committee (CCEA n°36) approved this study (no. 2023013113485359). Experiments were conducted in accordance with ARRIVE guidelines 2.0 for animal research reporting (Percie du Sert et al., 2020) and complied with the policies and regulations of *The Journal of Physiology* (O'Halloran, 2024). We used C57BL/6JRj male mice aged 8 weeks old (*n* = 8). The animal housing environment was maintained at 21 ± 1°C (mean ± SD) with a relative humidity of 55 ± 2%. Animals had access to food and water *ad libitum*. As previously described, we induce heart failure using TAC (Ghajar‐Rahimi et al., 2026) under deep anaesthesia (2.5% isoflurane mixed with room air). Following the recovery period, we injected the mice twice a day with buprenorphine (1 mg kg^−1^, s.c.) for further pain control. A minimally invasive deTAC procedure was performed when ejection fraction was measured below 45% (Ghajar‐Rahimi et al., 2026) and we injected the mice twice a day with buprenorphine (1 mg kg^−1^, s.c.) for further pain control. Animals were killed by cervical dislocation under deep anaesthesia (2.5% isoflurane in room air).

### Ultrasound imaging

A Vevo 3100 high‐frequency ultrasound system (FUJIFILM VisualSonics, Toronto, ON, Canada) with a 40 MHz centre frequency MX550D linear array probe was used to assess cardiac function on mice under isoflurane anaesthesia, as previously described (Ghajar‐Rahimi et al., 2026). We performed two‐dimensional (2D) echocardiography following American Physiological Society guidelines for assessment of murine LV cardiac function (Lindsey et al., 2018). The core temperature was maintained between 36 and 37.5°C, and the heart rate was monitored and maintained >450 beats min^−1^ throughout the procedure. We used the parasternal long‐axis (PLAX) view to collect B‐mode images of the left ventricle long‐axis view, parasternal short‐axis view at a frame rate of 250 frames s^−1^. We collected 4D left ventricle images in short‐axis view using a linear step motor (step size = 0.13 mm) to scan from the base of the apical epicardium to the ascending aorta to measure the mitral valve diameter at its maximal opening during diastole. If 4D ultrasound was unavailable we used a 2D short‐axis view collected at the LV base. Aortic valve diameter was measured from the PLAX view between the leaflets at their maximal systolic opening. All valve diameters were measured perpendicular to the direction of blood flow.

### Data processing and cycle definition

Offline images were used to calculate 2D LV strain and HDF analysis using Vevo Strain 2.0 (FUJIFILM VisualSonics), from the 2D PLAX B‐mode images.

Endocardial and myocardial boundaries were delineated at end‐systole and end‐diastole using Vevo Strain 2.0, with speckle‐tracking used to calculate intermediate boundary traces. The software‐placed end‐systole and end‐diastole markers were manually verified and, if necessary, corrected to align with the absolute maximum and minimum LV volumes, respectively.

The cardiac cycle was defined from the resultant LV volume curve. Systole was defined as the interval from the point of maximum LV volume (end‐diastole) to the first local minimum LV volume (end‐systole). Diastole comprises the interval from end‐systole to the subsequent end‐diastole.

### HDF component and interval definitions

HDF was resolved into longitudinal (apical–basal) and transverse (lateral–septal) components, assuming radial symmetry. The *z*‐axis was defined as originating at the LV apex (*z* = 0) and extending through the basal tissue between the mitral and aortic valves. Specific diastolic and systolic intervals were defined from the HDF curve and when possible, Root mean square (RMS) values of HDF metrics were calculated over their respective intervals to improve measurement accuracy. The selected HDF descriptors were chosen *a priori* based on published findings and their physiological interpretation (Arvidsson et al., 2018; Backhaus et al., 2022; Cesareo et al., 2024; Daal et al., 2022; Dal Ferro et al., 2019; Eriksson et al., 2016, 2017; Faganello et al., 2024; Li et al., 2025; Pedrizzetti et al., 2016, 2017; Pola et al., 2022, 2023; Sjöberg et al., 2018; Vairo et al., 2023). In particular, the systolic impulse based metrics reflect the active force generation during LV ejection under increased afterload and the diastolic E‐wave reflects desynchrony in left atrium and ventricle during early filling of the left ventricle. We define the following specific metrics to maintain consistent data collection across samples:

#### Diastolic E‐wave

This was defined as the interval during diastole commencing at the first positive‐going zero‐crossing of the HDF curve and concluding at the subsequent zero‐crossing. If the A‐wave did not produce a negative HDF value, the E‐wave was defined as ending at the inflection point between the E and A waves. If no A‐wave was present, the E‐wave extended to the end of diastole.

#### Systolic interval

The systolic peak was defined as the maximal HDF value during systole.

#### Systolic impulse

This was defined as the total interval from the start of systole (or first positive HDF value) until the HDF zero‐crossing immediately preceding end‐systole.

#### Early systolic impulse

This was defined as the interval from the HDF zero‐crossing (or systolic minimum) immediately preceding the systolic peak, up to the systolic peak.

#### Late systolic impulse

This was defined as the interval from the systolic peak to the end of the systolic impulse (the zero‐crossing immediately preceding end‐systole).

### Statistical analysis

All statistical analysis was performed with R version 4.5.1 (RStudio, Posit PBC, Boston, MA, USA) and the data are presented as mean ± SD. The Google Gemini Pro 3 model was used to generate select R functions for data analysis and code was manually verified. We used linear mixed‐effects modelling (LMM) to compare baseline, 3 week TAC and 4 week post‐deTAC global parameters. For interval timing metrics analysis, we used both the raw and normalized data for each timing metric to the overall RR interval length. We also used LMM to describe relationships between 3 week TAC and 4 week post‐deTAC variables.

Because HDF orientations represent directional data on a continuous 360° periodic scale, standard linear statistical methods are invalid. Consequently, all angular parameters were evaluated using dedicated circular statistics, adhering to the theoretical frameworks established by Mardia & Jupp (1999). Changes in the mean direction of HDF vectors between independent groups were assessed using the Watson–Williams multisample test. To specifically account for the longitudinal, repeated‐measures design of the TAC and deTAC interventions within the same subjects, the paired Watson–Williams test was utilized to evaluate intra‐subject angular shifts over time. All circular parameters are reported as the circular mean direction ± circular standard deviation.

To control the false discovery rate (FDR) associated with generating multiple exploratory HDF parameters, *P* values were adjusted using the Benjamini–Hochberg step‐up procedure. To prevent mathematical over‐penalization of inherently collinear variables, parameters were *a priori* divided into four distinct physiological families: Clinical Standards, Global HDF, Systolic HDF and Diastolic HDF. The FDR correction was applied independently within each functional family across the three longitudinal time points (Baseline, TAC, deTAC). Furthermore, normalized interval metrics (e.g. Normalized Systole Length) were strictly treated as mathematically redundant equivalents of their raw counterparts during the rank‐ordering phase of the FDR algorithm to preserve statistical power. An FDR‐adjusted *P* < 0.05 was considered statistically significant.

Principal component analysis (PCA) was run to determine new meaningful underlying variables, and included all cardiovascular metrics evaluated with high‐resolution ultrasound. We used a standardized PCA method because of the different measurement scales of the variables included. Principal components (PCs) were selected based on parallel analysis with 1000 simulations and a 95% percentile level. We plotted the first two PCs against one another to distinguish between the two groups. The contributions of a variable to PC1 and PC2 were averaged to evaluate the overall importance. We set the threshold for statistical significance at *P* < 0.05.

## Results

### LV systolic haemodynamic performance recovers in accordance with classical parameters

**Figure 1:**
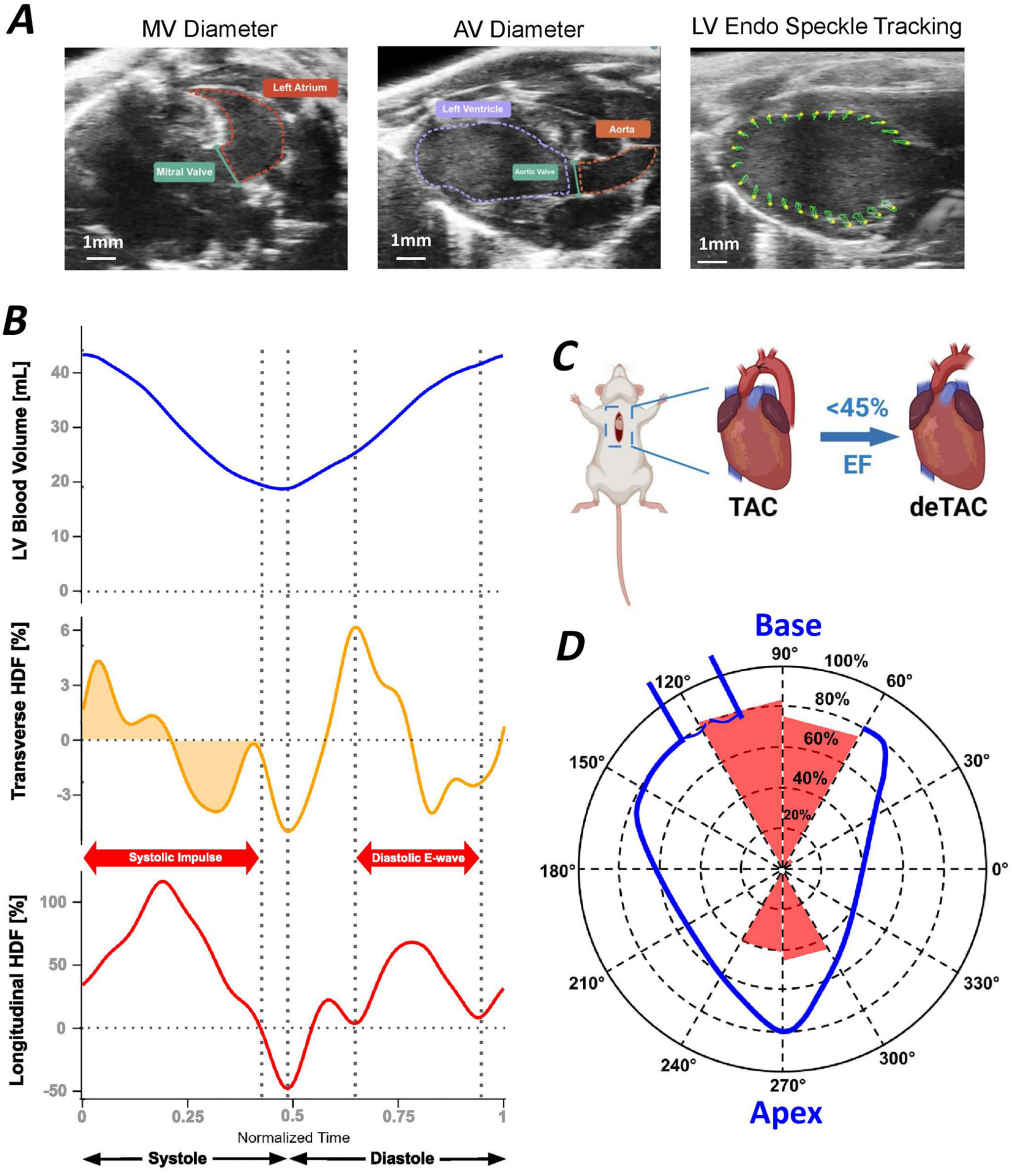
Echocardiographic characterization of left ventricular haemodynamic force (HDF) in a murine model *A*, labelled images of the three ultrasound scans used to determine the inputs for HDF calculation. From left to right, a short‐axis (SAX) view of the atrium for determining mitral valve (MV) diameter, a parasternal long‐axis (PLAX) view of the left ventricle to determine aortic valve (AV) diameter and another PLAX view of the left ventricle recorded over the cardiac cycle to conduct speckle tracking. *B*, representative curves of typical left ventricular (LV) volume (top, blue) and the corresponding transverse (middle, orange) and longitudinal (bottom, red) components of haemodynamic force plotted over a single, time‐normalized cardiac cycle. *C*, visual summary of the transverse aortic constriction (TAC) procedure followed by the debanding (deTAC) procedure. *D*, typical polar plot output from HDF calculations overlayed with an anatomical outline of the left ventricle in blue labelled with relevant reference points.

High‐resolution ultrasound was used to assess LV anatomical and functional trajectories at baseline, during TAC, and 4 weeks after deTAC (Fig. 1*A*–*C*; see also Appendix, Table A1). As previously described (Ghajar‐Rahimi et al., 2026; Salvas et al., 2026a), LV ejection fraction (LVEF) and LV longitudinal strain decreased during TAC and recovered after debanding (see Appendix, Table A1). To calculate HDF, we measured the mitral and aortic valves. Using 4D short‐axis scans at the base of the heart, we obtained a clear view of the mitral valve (Fig. 1*A*) and a 2D long‐axis view was used to measure the aortic valve. Although aortic valve area increased under TAC conditions before returning to baseline following debanding, mitral valve area remained stable (see Appendix, Table A1). During the period of this study, no animals died before the experiments were concluded, so there were no exclusions. Using Vevo Strain 2.0, we reconstructed full‐cycle HDF metrics, including transverse and longitudinal HDF (Fig. 1*B*), along with polar plots to assess HDF distribution in the left ventricle (Fig. 1*D*).

**Figure 2:**
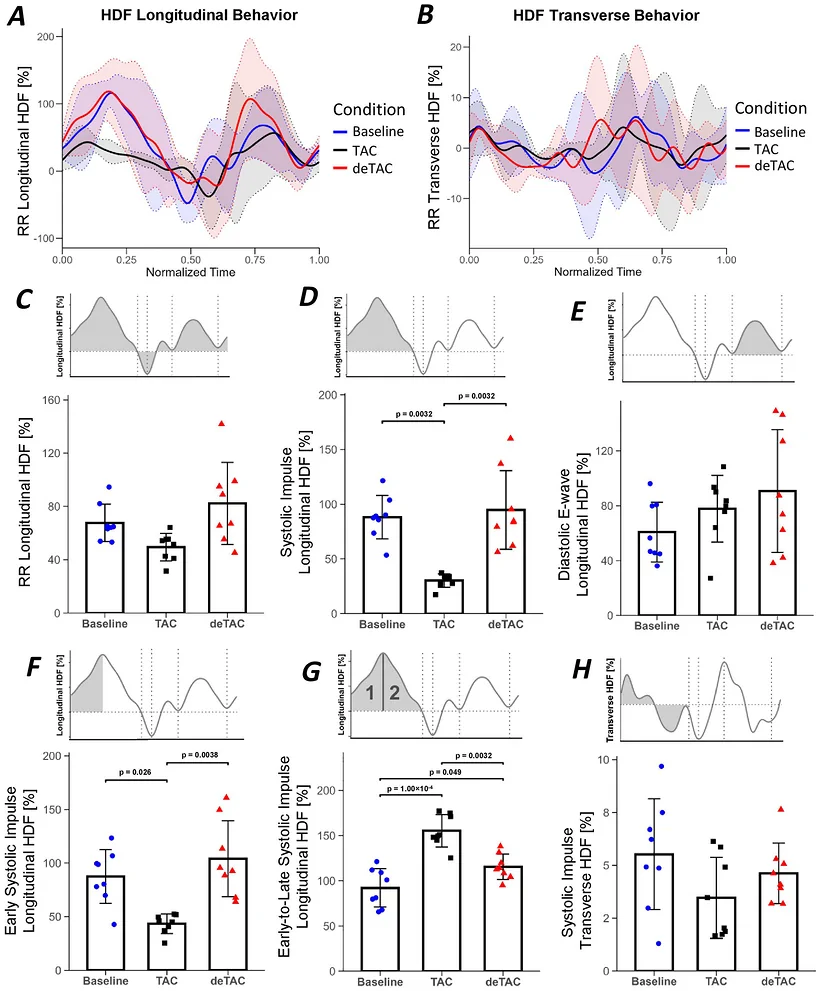
Phase‐specific and derived analysis of haemodynamic force (HDF) parameters in TAC/deTAC model *A*, mean longitudinal HDF behaviour over a normalized RR interval for baseline, TAC and deTAC conditions ± SD. *B*, mean transverse HDF behaviour over a normalized RR interval for baseline, TAC and deTAC conditions ± SD. *C*, longitudinal HDF across the entire RR interval; longitudinal baseline condition in blue, TAC condition in grey and deTAC condition in red. The trace above each graph represents the baseline mean graph behaviour with the region of interest highlighted. *D*, longitudinal HDF isolated to the systolic impulse phase, representing the primary force generation section of systole. *E*, diastolic E‐wave longitudinal HDF, representing the primary region of interest in the diastolic phase for HDF. *F*, early systolic impulse component of longitudinal HDF, representing a more specific interval of systolic force generation. *G*, a novel ratio of early‐to‐late systolic impulse longitudinal HDF, calculated by dividing the early systolic impulse (area labelled ‘1’) by the late systolic impulse (area labelled ‘2’). *H*, transverse HDF isolated to the systolic impulse phase, representing the vortex forming components in the left ventricle during systolic impulse.

Analysis of the overall longitudinal HDF profile (Figs 1*B* and 2*A* and *B*) shows that, under baseline conditions, forces rapidly rise at the onset of systole to a positive peak, followed by a gradual decline to a minimum marking the transition to diastole. During diastole, longitudinal forces increased again with minor fluctuations, displaying a distinct E‐wave peak and a final decrease during late diastolic filling (A‐wave). To quantify overall force amplitude, we compared the RMS values of longitudinal HDF across the entire R–R interval between baseline, TAC, and deTAC conditions (Fig. 2*C*). Unexpectedly, no significant difference was observed between groups for longitudinal HDF. Because global metrics are subject to specific interval with high variance, we assessed which phase of the cardiac cycle, systole or diastole, provides the most informative parameters to discriminate longitudinal HDF alterations (Fig. 2*D* and *E* and Table 1). The systolic impulse was significantly reduced in TAC hearts compared to both baseline and deTAC (*P* < 0.01) (Fig. 2*D*), whereas diastolic longitudinal HDF showed no significant differences among groups (Fig. 2*E*).

**Table 1:** HDF cardiac parameter intergroup comparisons using linear mixed effects and estimated marginal means in baseline, TAC and deTAC groups with physiological grouping and Benjamini–Hochberg correction applied

Metric	Baseline (mean ± SD)	TAC (mean ± SD)	deTAC (mean ± SD)	Baseline *vs*. TAC (p)	Baseline *vs*. deTAC (p)	TAC *vs*. deTAC (p)
RR length (ms)	120.88 ± 9.28	113.00 ± 13.98	116.00 ± 15.81	0.803	1	1
RR longitudinal RMS (%)	67.60 ± 14.04	49.44 ± 10.36	82.24 ± 30.82	0.684	0.684	0.24
RR transverse RMS (%)	7.04 ± 2.84	6.88 ± 3.01	7.05 ± 4.01	1	1	1
RR ratio RMS	10.55 ± 3.82	14.21 ± 6.44	8.53 ± 3.73	0.684	0.99	0.415
systole length (ms)	53.88 ± 6.29	58.38 ± 10.06	54.62 ± 15.75	0.91	0.996	0.949
Systolic impulse length (ms)	43.12 ± 8.03	50.75 ± 9.97	45.62 ± 10.20	0.457	0.974	0.729
Early systolic impulse length (ms)	16.88 ± 5.41	9.12 ± 2.42	20.75 ± 6.69	0.053	0.498	0.0050
Late systolic impulse length (ms)	24.12 ± 5.96	39.12 ± 7.59	23.25 ± 5.28	0.0032**	0.996	0.003**
Systole length normalized	44.68 ± 5.09	52.12 ± 10.02	47.09 ± 11.26	0.418	0.952	0.680
Systolic impulse length normalized	35.85 ± 7.09	45.32 ± 9.76	39.60 ± 8.10	0.183	0.796	0.559
Early systolic impulse length normalized	14.06 ± 4.49	8.18 ± 2.46	17.83 ± 5.20	0.080	0.338	0.0046
Late systolic impulse length normalized	19.96 ± 4.78	34.85 ± 7.01	20.51 ± 6.56	0.0036**	0.996	0.0038**
Early‐to‐late systolic impulse length ratio	76.90 ± 40.24	24.39 ± 9.05	94.98 ± 39.82	0.044*	0.729	0.0074**
Systolic impulse longitudinal RMS (%)	88.15 ± 19.95	30.10 ± 6.15	94.75 ± 35.95	0.0032**	0.974	0.0032**
Systolic impulse transverse RMS (%)	5.53 ± 2.62	3.46 ± 1.92	4.62 ± 1.43	0.267	0.870	0.729
Systolic impulse AUC (%∙ms)	31.91 ± 11.83	4.70 ± 2.04	31.35 ± 22.20	0.0102*	0.996	0.0106*
Systolic peak (%)	129.17 ± 28.89	55.92 ± 10.03	141.51 ± 58.55	0.0102	0.949	0.0038
Early systolic impulse longitudinal RMS (%)	87.47 ± 25.04	43.45 ± 9.05	104.10 ± 35.42	0.026*	0.627	0.0038**
Late systolic impulse longitudinal RMS (%)	95.98 ± 26.72	28.02 ± 5.34	91.76 ± 37.38	0.0032**	0.996	0.0032**
Late systole longitudinal RMS (%)	41.25 ± 15.55	19.96 ± 19.94	37.67 ± 11.86	0.1008	0.993	0.202
Early‐to‐late systolic impulse longitudinal ratio	92.22 ± 21.12	155.31 ± 17.95	115.50 ± 14.09	1.00 × 10^−4^***	0.049*	0.0032**
E‐wave longitudinal RMS (%)	60.81 ± 21.77	77.81 ± 24.27	90.74 ± 44.80	0.854	0.854	0.899
E‐wave ratio RMS	12.24 ± 8.02	12.74 ± 6.32	9.10 ± 7.30	0.985	0.854	0.854

Statistical notations: *P ≤ 0.05, **P ≤ 0.01 and ***P ≤ 0.001.

Given these findings, we focused on a detailed analysis of systolic dynamics by calculating more than fifteen systolic force parameters (Table 1). Among these parameters, the early systolic impulse RMS of longitudinal HDF was significantly reduced in TAC hearts compared to both baseline and deTAC hearts (*P* < 0.05 and *P* < 0.01) (Fig. 2*F*). Interestingly, the early‐to‐late systolic impulse longitudinal force ratio was significantly increased during TAC compared to baseline and deTAC (*P* < 0.01) (Fig. 2*G*). Notably, this ratio did not return to baseline values after deTAC and remained significantly elevated relative to baseline (*P* < 0.05). Finally, analysis of systolic transverse impulse parameters revealed no significant differences between groups (Fig. 2*B* and *H* and Table 1).

### Interval‐specific haemodynamic forces show promise for improved correlative tracking of ventricular remodelling compared to strain

**Figure 3:**
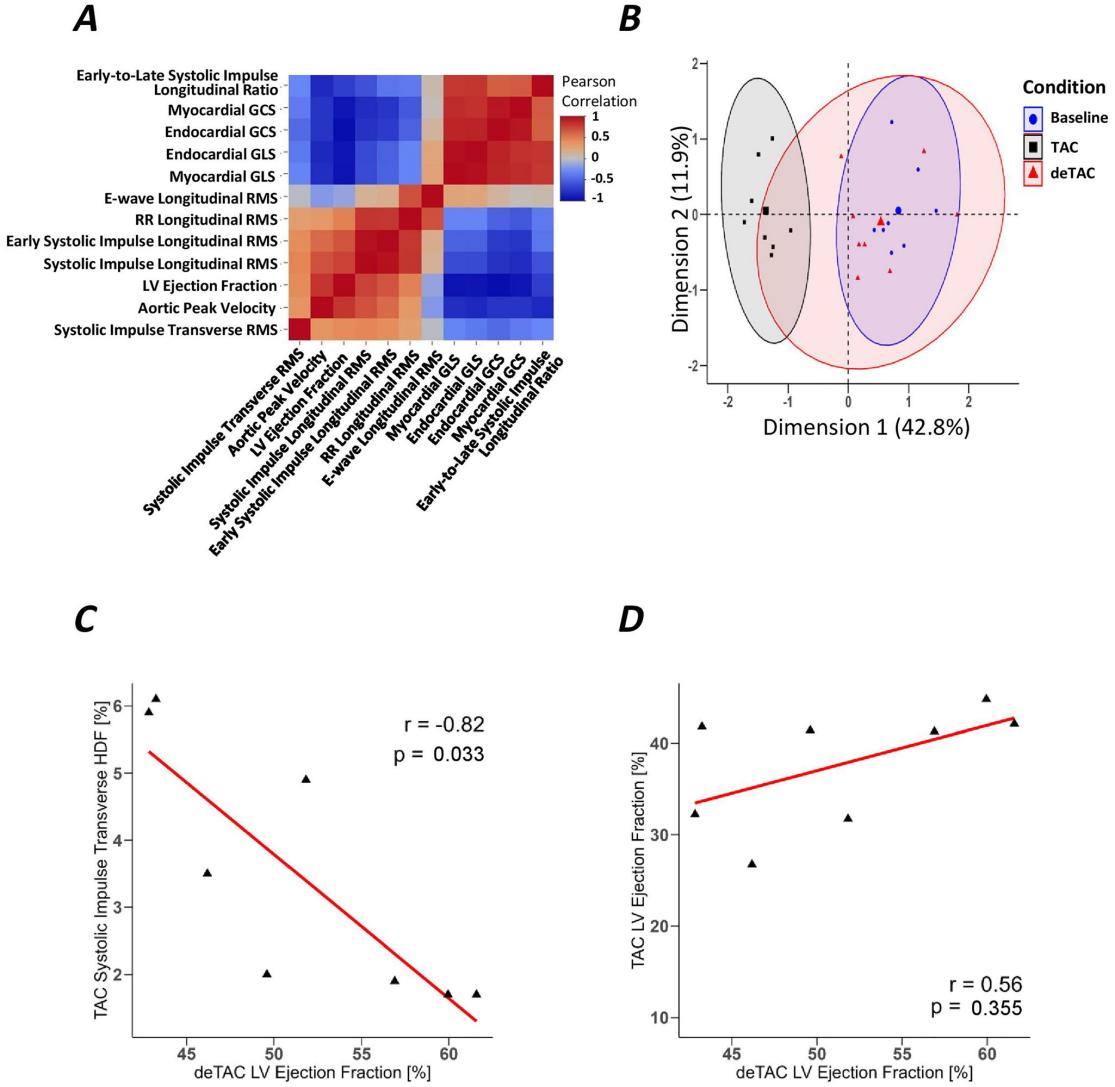
Correlative analysis of HDF and traditional echocardiographic parameters *A*, intercorrelation matrix of strain, ejection fraction, and aortic peak velocity against various HDF parameters. The colour scale indicates the strength and direction of the correlation, where deep red signifies a strong positive correlation (*r* ≈ 1) and deep blue signifies a strong negative correlation (*r* ≈ –1). The distinct colour blocks demonstrate that strain parameters and the derived HDF ratio metric are highly correlated with one another, as well as that ejection fraction and aortic peak velocity are highly correlated with longitudinal HDF parameters. *B*, partial component analysis (PCA) of individual mouse samples across all metrics measured. The high variability of the deTAC condition demonstrates how broader and many diastolic HDF parameters do not show recovery after deTAC. *C*, grouped correlation of TAC condition systolic impulse transverse HDF against deTAC LVEF recovery (*r* = −0.82, *P* = 0.033). *D*, grouped correlation of TAC condition LVEF against deTAC LVEF recovery (*r* = 0.56, *P* = 0.355).

**Figure A1:**
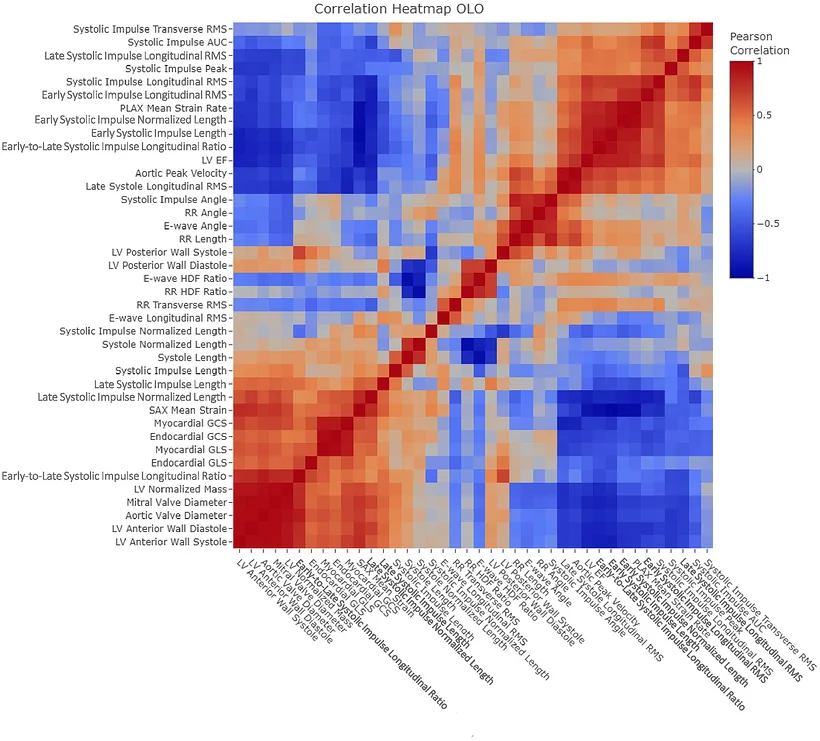
Overall correlation behaviour of metrics of interest are summarized in the intercorrelation matrix The colour scale indicates the strength and direction of the correlation, where deep red signifies a strong positive correlation (*r* ≈ 1) and deep blue signifies a strong negative correlation (*r* ≈ −1).

We then assessed the degree to which HDF metrics correlate with classical cardiovascular function, such as ejection fraction, LV strain and aortic peak velocity, by conducting linear mixed‐effects correlation tests. The overall correlation behaviour of metrics of interest are summarized in the intercorrelation matrix (see Appendix, Fig. A1), which identifies two distinct sets of groups in the clustered blue and red regions (Fig. 3*A*). Most HDF metrics show a positive correlation with LVEF and aortic peak velocity, except for the E‐wave longitudinal RMS, which behaves unpredictably, and the early‐to‐late systolic impulse longitudinal force ratio, which shows a predictable but inverse relationship (Fig. 3*A*). Interestingly, transverse component of the systolic impulse, which was not significant in intergroup comparisons was found to be significant for the paired TAC metric to deTAC LVEF (*r* = −0.82, *P* = 0.033; see also Appendix, Table A2) when conducting linear mixed‐effects correlation tests (Fig. 3*D*). HDF angle was tested at systolic and diastolic intervals, and despite what previous studies have found in humans (Cesareo et al., 2024; Pedrizetti et al., 2016), HDF angle was non‐significant in all cases (Table 2).

**Table 2:** HDF angle parameter intergroup comparisons using paired Watson–Williams test and estimated marginal means in baseline, TAC and deTAC groups with physiological grouping and Benjamini–Hochberg correction applied

Metric	Baseline (mean ± SD)	TAC (mean ± SD)	deTAC (mean ± SD)	Baseline *vs*. TAC (*P*)	Baseline *vs*. deTAC (*P*)	TAC *vs*. deTAC (*P*)
RR angle (°)	80.50 ± 3.38	78.75 ± 3.88	82.00 ± 3.02	0.684	0.684	0.415
Systolic impulse angle (°)	83.75 ± 3.11	81.25 ± 5.99	85.38 ± 1.06	0.498	0.320	0.152
E‐wave angle (°)	81.75 ± 4.46	79.62 ± 4.41	82.00 ± 6.05	0.854	0.985	0.854

Statistical notations: *P ≤ 0.05, **P ≤ 0.01 and ***P ≤ 0.001.

**Figure A2:**
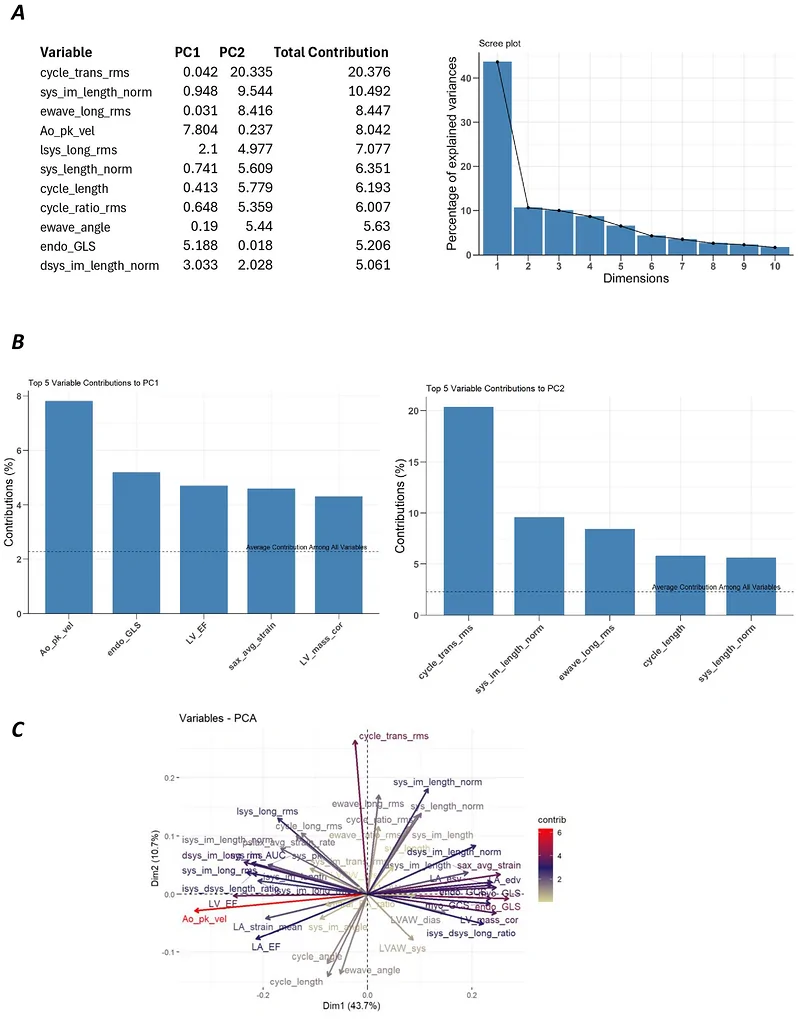
Principal component analysis *A*–*C*, principal component analysis showing the variables contributing to the first two principal components.

PCA was then applied to test whether combined HDF and functional metrics could discriminate between experimental conditions. The first two principal components explained 55% of the total variance (PC1: 43%, PC2: 12%) (see Appendix,Fig. A2*A*), with baseline and TAC groups showing partial separation along PC1 and deTAC animals occupying an intermediate position, consistent with incomplete reversal of the TAC‐induced alterations (Fig. 3*B*). PC1 was mainly driven by aortic peak velocity, endocardial global longitudinal strain (GLS), LVEF and systolic strain indices, and therefore reflected a global systolic function/loading axis with TAC hearts at one extreme, baseline at the opposite and deTAC in between. By contrast, PC2 was dominated by transverse HDF amplitude (cycle_trans_rms), systolic longitudinal HDF length (sys_im_length_norm), E‐wave longitudinal HDF (e_wave_long_rms) and cycle length (cycle_length). This captured a haemodynamic force pattern/timing axis that separated animals by transverse force magnitude, spatial extent and cycle duration, not conventional systolic indices (Fig. A2*A*–*C*). TAC induced a distinct multivariate HDF signature, only partially normalized after debanding and missed by univariate parameters. Finally, we assessed whether any HDF or conventional metrics measured during TAC showed associations with the reversal of LV functional impairment after debanding. Using linear mixed‐effects correlation analysis (see Appendix,Tables A2, A3, A4), we found that, among the 41 parameters tested, only HDF‐derived metrics, specifically the systolic transverse RMS and systolic transverse impulse, correlate with reverse LV functional recovery (Fig. 3*C*), whereas ejection fraction failed to show any predictive value (Fig. 3*D*).

## Discussion

Our preclinical study demonstrates that HDF parameters derived from high‐resolution ultrasound reliably track cardiac function and closely align with established markers such as GLS, circumferential strain, and LVEF. Beyond this concordance, our findings indicate that HDF metrics offer added value by identifying candidate markers of LV remodelling and functional recovery following surgical intervention. Together, these results highlight the potential of HDF analysis to enhance prognostic assessment of LV recovery beyond conventional echocardiographic measures.

Recent advances in ultrasound and cardiac imaging analysis methodologies have rapidly expanded the tools available to clinicians and researchers, enabling deeper, faster and more accurate assessment of cardiac function during disease progression (Gillam & Marcoff, 2024). Beyond conventional measures such as ejection fraction, segmental cardiac strain has markedly improved the early detection of developing heart failure following myocardial infarction or pressure overload, including aortic constriction, by revealing subtle patterns of myocardial deformation abnormalities (Smiseth et al., 2025). Initially developed for clinical applications, cardiac strain analysis has now been incorporated into recent guidelines for assessing cardiac function in rodent models (Lindsey et al., 2018; Zacchigna et al., 2020). Because LV function cannot be fully captured by ejection fraction alone, the heart must be considered as an integrated system in which cavity interdependence plays a critical role. Consequently, holistic approaches could be used to better understand cardiac dysfunction trajectory, including the assessment of left atrial functional dynamics (Salvas et al., 2025b). More recently, the interaction between myocardial motion and intracardiac fluid dynamics, assessed by cardiac MRI and increasingly by advanced ultrasound techniques, has gained attention. Early evaluation of HDF has been shown to carry prognostic value in asymptomatic patients and may provide unique insights into myocardial dysfunction that extend beyond traditional echocardiographic parameters and deformation‐based measures (Vallelonga et al., 2021). Unlike global strain or LVEF, which summarize deformation or volume change, systolic HDF evaluate the magnitude and orientation of intraventricular pressure gradients, so that early maladaptive remodelling appears as ‘misaligned’ or more transverse forces even when strain and ejection fraction are still within preserved or only mildly reduced ranges (Backhaus et al., 2022). Specifically, HDF analysis has been shown to differentiate patients who recover LV function after transcatheter aortic valve implantation (TAVI) from those with persistent haemodynamic dysfunction (Angotti et al., 2025; Vairo et al., 2023). In this preclinical study, we successfully applied ultrasound‐based HDF analysis in a mouse model of aortic banding and debanding that mimics the treatment of aortic stenosis. Following debanding, systolic HDF components and overall LV longitudinal force improved significantly, indicating that recovery of contractile function is accompanied by a marked normalization of intraventricular fluid dynamics (Angotti et al., 2025; Vairo et al., 2023). Although strain and ejection fraction metrics correlate well with aortic peak velocity, an established indicator of LV systolic performance, our findings suggest that, compared with systolic HDF components, these conventional metrics have less predictive power when used as single time‐point measurements to anticipate functional recovery after surgical intervention. We tested for any specific variation in effects for diastolic and systolic regions of interest. In particular, previous reports have found that the regions of interest are the systolic impulse (Faganello et al., 2024; Li et al., 2025) and diastolic E‐wave regions (Backhaus et al., 2022; Eriksson et al., 2016). In this TAC and deTAC model, systolic impulse metrics were generally found to have much higher significance as compared to the diastolic E‐wave and HDF angle metrics. Furthermore, the more specified the intervals became, the more significant that the intergroup statistical power became. These findings highlight the particular value of temporally and spatially resolved systolic HDF descriptors in characterizing disease severity and reversibility in this model. Consistent with recent 4D flow phase contrast MRI‐based studies indicating that the prognostic value of current HDF implementations in heart failure with preserved ejection fraction (HFpEF) depends strongly on parameter selection and analytical context, our data support the need for careful metric choice and contextual interpretation to extract robust prognostic information from HDF analysis (Arvidsson et al., 2022). Building on this, PCA of combined HDF and conventional echocardiographic variables revealed that systolic HDF features contributed most strongly to the main axis separating baseline, TAC and deTAC conditions, whereas diastolic E‑wave and HDF angle related metrics had only modest influence on group discrimination. The data thus reflects the high variability observed during diastole, as seen in both longitudinal and transverse HDF components. This suggests that, at least in the context of pressure overload and its relief, altered systolic force generation and turbulent flow within the ventricle capture mechanisms driving maladaptive remodelling, whereas diastolic HDF changes may be subtler, secondary phenomena. Focusing on carefully defined systolic windows therefore appears to enhance both the sensitivity and the statistical power of HDF‐based analyses, supporting their use as potential early integrative markers of LV contractile recovery after unloading interventions (Angotti et al., 2025; Vairo et al., 2023; Vallelonga et al., 2021). Taken together, these results suggest that echocardiographic assessment of HDFs may provide a tool to distinguish responders from non‐responders after surgical treatment, thereby identifying individuals at risk of persistent haemodynamic dysfunction.

### Limitations

The use of 2D speckle‐tracking echocardiography in Vevo Strain 2.0 for HDF calculations relies on assumptions from a single PLAX view, which cannot capture true 3D myocardial motion including out‐of‐plane rotation and deformation, leading to underestimation of transverse strain components; transitioning to 4D HDF analysis could enhance reproducibility by enabling full volumetric tracking from a single dataset, at the same time as potentially revealing finer details such as diastolic E‐wave parameters and angle variations that exhibit high variability in 2D assessments. Despite variability, it is possible that the apparent robustness of HDF metrics could arise at least in part from the spatial and temporal averaging of 2D measurements used for integration. Although the use of HDF metrics shows theoretical benefits over Euler IVPG, we did not assess eccentric remodelling (Opie et al., 2006) or a comparison against doppler‐derived traditional Euler IVPGs, and so a direct benefit is not explored in this study. Furthermore, because HDF is a closely related momentum‐balance calculation to IVPG, we acknowledge that it should not be interpreted as a fundamentally independent physiological metric.

As a result of the small study cohort (*n* = 8), these multivariate analyses are mainly hypothesis‐generating and are not necessarily reflective of a causative relationship. Instead, our PCA analysis was used to summarize overall structure of our dataset and help identify correlative relationships.

Additionally, our analysis emphasized systolic HDF metrics as a result of their superior statistical power and predictive value in this TAC/deTAC model, potentially overlooking diastolic E‐wave contributions that could offer complementary insights into filling dynamics and vary across different heart failure etiologies. Future work will be needed to create analysis strategies that fully combine a wider variety of metrics to fully characterize cardiac remodelling.

## Conclusions

This preclinical study establishes ultrasound‐derived HDF metrics, particularly systolic transverse impulse components, as possible predictors of LV functional recovery following aortic debanding in a mouse TAC model, establishing baseline performance in line with conventional strain and ejection fraction measures at single time points at the same time as laying the groundwork for further work to show potential advantages of the metric. These findings underscore the value of integrating intraventricular fluid dynamics with myocardial deformation for early identification of responders *vs*. non‐responders to unloading interventions. Although future investigations incorporating 3D/4D HDF analysis, diastolic‐focused assessments, direct comparison against doppler‐derived IVPG measurements and sex‐balanced cohorts could refine their clinical translatability in pressure overload heart failure, this work demonstrates the potential of HDF waveform analysis to improve assessment of LV remodelling over standard echocardiographic measures.

## Additional information

## Competing interests

All authors have read the journal's policy on disclosure of potential conflicts of interest. C. J. Goergen serves on the Scientific Advisory Board for FUJIFILM VisualSonics Inc. None of the other authors have conflicts of interest, financial or otherwise, to disclose. Disclaimers: FUJIFILM VisualSonics Inc. had no role in the study's design, execution, interpretation or writing.

## Author contributions

P.D. was responsible for formal analysis. P.D., T.M.‐M. and P.S. were responsible for investigations. P.D. was responsible for visualization. P.D., T.M.‐M. and P.S. were responsible for writing the original draft. P.D., T.M.‐M., C.J.G. and P.S. were responsible for responsible for reviewing and editing. T.M.‐M., C.J.G. and P.S. were responsible for conceptualization. T.M.‐M., C.J.G. and P.S. were responsible for methodology. T.M.‐M., C.J.G. and P.S. were responsible for supervision.

## Funding

This study is funded by Fulbright U.S. Scholar Program (CJG), Thomas Jefferson Fund from the French‐American Cultural Exchange Foundation (CJG, PS), Biocampus (PS) and I‐Site Muse (PS).

## Generative AI statement

The Google Gemini Pro 3 model was used to generate select R functions for data analysis and code was manually verified. This was the only generative AI used in the preparation of this manuscript.

## Supporting information


Peer Review History

## Data Availability

The data that support the findings of this study are available from the corresponding author upon reasonable request.

## Appendix

**Table A1:** Traditional cardiac parameter intergroup comparisons using linear mixed effects and estimated marginal means in baseline, TAC and deTAC groups with physiological grouping and Benjamini–Hochberg correction applied

Metric	Baseline (mean ± SD)	TAC (mean ± SD)	deTAC (mean ± SD)	Baseline *vs*. TAC (*P*)	Baseline *vs*. deTAC (*P*)	TAC *vs*. deTAC (*P*)
Aortic peak velocity (mm s^−1^)	−979.86 ± 121.04	−3737.59 ± 396.37	−1639.79 ± 311.64	1.75 × 10^−9^***	0.0026**	2.69 × 10^−8^***
Mitral valve E‐wave to A‐wave ratio	1.89 ± 0.77	1.33 ± 0.88	1.49 ± 0.60	0.346	0.601	0.992
LV EF (%)	58.36 ± 4.94	37.77 ± 6.54	51.52 ± 7.34	0.0001***	0.153	0.0026**
Mitral valve diameter (mm)	1.64 ± 0.04	1.74 ± 0.11	1.66 ± 0.17	0.273	0.992	0.404
Aortic valve diameter (mm)	1.26 ± 0.04	1.38 ± 0.13	1.25 ± 0.10	0.072	0.992	0.043*
Myocardial GCS (%)	−15.13 ± 1.96	−9.85 ± 1.92	−14.87 ± 2.38	0.0011**	0.992	0.0017**
Myocardial GLS (%)	−15.46 ± 1.92	−7.40 ± 2.38	−11.58 ± 2.79	4.01 × 10^−5^***	0.012*	0.0018**
Endocardial GCS (%)	−27.67 ± 3.69	−17.00 ± 3.34	−24.93 ± 4.54	0.0011**	0.481	0.0069**
Endocardial GLS (%)	−21.46 ± 2.38	−10.32 ± 2.80	−16.71 ± 3.62	1.86 × 10^−5^***	0.014*	0.0018**
PLAX mean strain rate (d%/dt)	7.41 ± 1.99	5.59 ± 1.83	7.56 ± 2.45	0.355	0.992	0.303
SAX mean strain (%)	−29.17 ± 2.37	−16.94 ± 3.73	−26.29 ± 5.69	0.0001***	0.404	0.0017**
LV normalized mass (mg)	84.09 ± 15.74	148.84 ± 11.81	105.93 ± 29.61	0.0004***	0.222	0.073
LV anterior wall diastole (mm)	0.88 ± 0.24	1.21 ± 0.17	0.94 ± 0.14	0.017*	0.942	0.062
LV posterior wall diastole (mm)	0.77 ± 0.27	0.95 ± 0.14	0.76 ± 0.06	0.231	0.992	0.180
LV anterior wall systole (mm)	1.36 ± 0.30	1.54 ± 0.25	1.38 ± 0.36	0.684	0.992	0.759
LV posterior wall systole (mm)	1.18 ± 0.21	1.14 ± 0.17	1.12 ± 0.10	0.992	0.933	0.992

Statistical notations: **P* ≤ 0.05, ***P* ≤ 0.01 and ****P* ≤ 0.001.

**Table A2:** Linear mixed effects correlation of HDF cardiac parameters against left ventricular ejection fraction across all conditions and paired−conditions of TAC metrics to deTAC left ventricular ejection fraction with physiological grouping and Benjamini–Hochberg correction applied

Metric	Correlation between all groups (*n* = 24)		Correlation TAC metrics *vs*. deTAC LVEF (*n* = 8)	
	*r* value	*P* value	*r* value	*P* value
RR length	0.41	0.240	0.47	0.488
Systole length	−0.04	0.888	0.71	0.110
Systolic impulse length	−0.14	0.700	0.64	0.191
Early systolic impulse length	0.58	0.011*	0.31	0.622
Late systolic impulse length	−0.58	0.011*	0.43	0.455
Systole length normalized	−0.29	0.307	0.40	0.482
Systolic impulse length normalized	−0.34	0.202	0.37	0.531
Early systolic impulse length normalized	0.52	0.027*	0.12	0.844
Late systolic impulse length normalized	−0.67	0.0019**	0.17	0.806
Early‐to‐late systolic impulse length ratio	0.65	0.0027**	−0.03	0.973
RR longitudinal RMS	0.47	0.210	0.02	0.968
RR transverse RMS	0.07	0.968	0.02	0.968
RR ratio RMS	−0.35	0.310	0.04	0.968
Systolic impulse longitudinal RMS	0.75	0.0003***	0.00	0.994
Systolic impulse transverse RMS	0.42	0.110	−0.82	0.033*
Systolic impulse AUC	0.80	0.0001***	0.53	0.307
Systolic peak	0.72	0.0007***	0.24	0.732
Early systolic impulse longitudinal RMS	0.67	0.0019**	−0.17	0.806
Late systolic impulse longitudinal RMS	0.77	0.0002***	0.15	0.817
Late systole longitudinal RMS	0.57	0.0131*	0.18	0.806
Early‐to‐late systolic impulse longitudinal ratio	−0.71	0.0008***	−0.46	0.422
E‐wave longitudinal RMS	−0.15	0.726	−0.06	0.881
E‐wave ratio RMS	−0.08	0.874	0.43	0.670

Statistical notations: **P* ≤ 0.05, ***P* ≤ 0.01 and ****P* ≤ 0.001.

**Table A3:** Mardia‐Jupp method correlation of HDF angle parameters against left ventricular ejection fraction across all conditions and paired‐conditions of TAC metrics to deTAC left ventricular ejection fraction with physiological grouping and Benjamini–Hochberg correction applied

Metric	Correlation between all groups (*n* = 24)		Correlation TAC metrics *vs*. deTAC LVEF (*n* = 8)	
	*r* value	*P* value	*r* value	*P* value
RR angle	0.31	0.968	0.60	0.488
Systolic impulse angle	0.47	0.307	0.84	0.110
E‐wave angle	0.35	0.624	0.66	0.670

Statistical notations: **P* ≤ 0.05, ***P* ≤ 0.01 and ****P* ≤ 0.001.

**Table A4:** Pearson correlation of traditional cardiac parameters against left ventricular ejection fraction across all conditions and paired‐conditions of TAC metrics to deTAC left ventricular ejection fraction with physiological grouping and Benjamini–Hochberg correction applied

Metric	Correlation between all groups (*n* = 24)		Correlation TAC metrics *vs*. deTAC LVEF fraction (*n* = 8)	
	*r* value	*P* value	*r* value	*P* value
Aortic peak velocity	0.80	2.04 × 10^−5^***	0.18	0.753
Mitral valve E‐wave to A‐wave ratio	0.17	0.561	−0.46	0.433
LV EF	NA	NA	0.56	0.355
Myocardial GCS	−0.93	2.75 × 10^−10^***	−0.65	0.235
Myocardial GLS	−0.94	2.63 × 10^−10^***	−0.50	0.399
Endocardial GCS	−0.98	2.75 × 10^−15^***	−0.54	0.358
Endocardial GLS	−0.94	2.75 × 10^−10^***	−0.49	0.399
PLAX mean strain rate	0.50	0.042*	0.02	0.995
SAX mean strain	−0.88	5.07 × 10^−8^***	−0.18	0.753
LV normalized mass	−0.75	3.73 × 10^−5^***	−0.276	0.647
LV anterior wall diastole	−0.48	0.045*	0.450	0.433
LV posterior wall diastole	−0.32	0.318	−0.050	0.977
LV anterior wall systole	−0.19	0.521	0.422	0.438
LV posterior wall systole	0.22	0.438	0.234	0.702

Statistical notations: **P* ≤ 0.05, ***P* ≤ 0.01 and ****P* ≤ 0.001. NA, not applicable.
