# Supplementary material for: Ultrasound‐based haemodynamic force trajectories in a murine model of reversible pressure overload
Source: J Physiol. 2026 Jul 23;604(17):7092–111. doi: 10.1113/JP290877 (PMC13532889; doi:10.1113/JP290877)
Supplement: Supplementary file 1 [file TJP-604-7092-s001.pdf]

The Journal of Physiology

# Ultrasound-Based Hemodynamic Force Trajectories in a Murine Model of Reversible Pressure Overload

Peyton Day, Thomas Moore-Morris, Craig J Goergen, and Pierre Sicard
**DOI: 10.1113/JP290877**

*Corresponding author(s): Pierre Sicard (pierre.sicard@inserm.fr)*

*The referees have opted to remain anonymous.*



## Review Timeline:

| | |
|---|---|
| Submission Date: | 07-Jan-2026 |
| Editorial Decision: | 16-Feb-2026 |
| Revision Received: | 02-May-2026 |
| Editorial Decision: | 08-Jun-2026 |
| Revision Received: | 17-Jun-2026 |
| Accepted: | 24-Jun-2026 |

*Senior Editor: Natalia Trayanova*

*Reviewing Editor: Ana Simón-Chica*


## Transaction Report:

(Note: With the exception of the correction of typographical or spelling errors that could be a source of ambiguity, letters and reports are not edited. Depending on transfer agreements, referee reports obtained elsewhere may or may not be included in this compilation. Referee reports are anonymous unless the Referee chooses to sign their reports.)

Re: JP-RP-2026-290877 "**Ultrasound-Based Hemodynamic Force Trajectories in a Murine Model of Reversible Pressure Overload**" by Peyton Day, Thomas Moore-Morris, Craig J Goergen, and Pierre Sicard

Dear Dr Sicard,

Thank you for submitting your manuscript to The Journal of Physiology. It has been assessed by a Reviewing Editor and by 2 expert referees and we are pleased to tell you that it is potentially acceptable for publication following satisfactory major revision.

Please advise your co-authors of this decision as soon as possible.

The referee reports are copied at the end of this email.

Please address all the points raised and incorporate all requested revisions or explain in your Response to Referees why a change has not been made. We hope you will find the comments helpful and that you will be able to return your revised manuscript within 2 months. If your article is NOT for a Special Issue, you may have 9 months to revise. If you require an extension, please contact journal staff: jp@physoc.org. Please note that this letter does not constitute a guarantee for acceptance of your revised manuscript.

Your revised manuscript should be submitted online using the link in your Author Tasks: Link Not Available. This link is accessible via your account as Corresponding Author; it is not available to your co-authors. If this presents a problem, please contact journal staff (jp@physoc.org). Image files from the previous version are retained on the system. Please ensure you replace or remove any files that are being revised.

If you do not wish to submit a revised version of your manuscript, you must inform our journal staff (jp@physoc.org) or reply to this email to request withdrawal. Please note that a manuscript must be formally withdrawn from the peer review process at one journal before it may be submitted to another journal.

TRANSPARENT PEER REVIEW POLICY: To improve the transparency of its peer review process, The Journal of Physiology publishes online as supporting information the peer review history of all articles accepted for publication. Readers will have access to decision letters, including Editors' comments and referee reports, for each version of the manuscript, as well as any author responses to peer review comments. Referees can decide whether or not they wish to be named on the peer review history document.

ABSTRACT FIGURES: Authors are expected to use The Journal's premium BioRender account to create/redraw their Abstract Figures. Information on how to access this account is here: https://physoc.onlinelibrary.wiley.com/journal/14697793/biorender-access.

This will enable Authors to create and download high-resolution figures. If authors have used the free BioRender service, they can use the instructions provided in the link above to download a high-resolution version suitable for publication.

The link provided should only be used for the purposes of this submission. Authors will be charged for figures created on this account if they are not related to this manuscript submission.

LANGUAGE EDITING AND SUPPORT FOR PUBLICATION: If you would like help with English language editing, or other article preparation support, Wiley Editing Services offers expert help, including English Language Editing, as well as translation, manuscript formatting, and figure formatting at www.wileyauthors.com/eeo/preparation. You can also find resources for Preparing Your Article for general guidance about writing and preparing your manuscript at www.wileyauthors.com/eeo/prepresources.

RIGOUR AND REPRODUCIBILITY: We recommend authors consult The Journal's Rigour and Reproducibility requirements to ensure that their manuscript is suitable for publication: https://physoc.onlinelibrary.wiley.com/pb-assets/hub-assets/physoc/documents/TJP-Rigour-and-Reproducibility-Requirements.pdf

REVISION CHECKLIST:

Check that your Methods section conforms to journal policy: https://jp.msubmit.net/cgi-bin/main.plex?form_type=display_requirements#methods.

Check that data presented conforms to the statistics policy: https://jp.msubmit.net/cgi-bin/main.plex?form_type=display_requirements#statistics.

Upload a full Response to Referees file. To create your 'Response to Referees': copy all the reports, including any comments from the Senior and Reviewing Editors, into a Microsoft Word, or similar, file and respond to each point, using font or background colour to distinguish comments and responses and upload as the required file type.

Please upload two versions of your manuscript text: one with all relevant changes highlighted and one clean version with no changes tracked. The manuscript file should include all tables and figure legends, but each figure/graph should be uploaded as separate, high-resolution files.

You may also upload:

- 'Potential Cover Art' for consideration as the issue's cover image
- Appropriate Supporting Information (Video, audio or data set: see https://jp.msubmit.net/cgi-bin/main.plex?form_type=display_requirements#supp).

We look forward to receiving your revised submission.

If you have any queries, please reply to this email and we will be pleased to advise.

Yours sincerely,

Natalia Trayanova
Senior Editor
The Journal of Physiology

----------------

REQUIRED ITEMS

- Author photo and profile. First or joint first authors are asked to provide a short biography (no more than 100 words for one author or 150 words in total for joint first authors) and a portrait photograph. These should be uploaded and clearly labelled together in a Word document with the revised version of the manuscript. See Information for Authors for further details.

- The contact information for the person responsible for 'Research Governance' at your institution needs to be provided. This includes their name and an institutional email address. Please ensure the contact is not an author on this paper and provide an alternate contact if necessary, or confirm in the submission form that the author whose email was provided has sole responsibility for research governance. This is the person who is responsible for regulations, principles and standards of good practice in research carried out at the institution, for instance the ethical treatment of animals, the keeping of proper experimental records or the reporting of results.

- You must start the Methods section with a paragraph headed Ethical approval (https://jp.msubmit.net/cgi-bin/main.plex?form_type=display_requirements#methods).

Research must comply with The Journal's policies regarding animal experiments (https://physoc.onlinelibrary.wiley.com/hub/animal-experiments) and adherence to these policies must be stated in the manuscript.

Authors should confirm in their Methods section that their experiments were carried out according to the guidelines laid down by their institution's animal welfare committee, including an ethics approval reference number. The Methods section must contain a statement about access to food, water and housing, details of the anaesthetic regime: anaesthetic used, dose and route of administration, and method of killing the experimental animals.

- Please upload separate high-quality figure files via the submission form.

- Please ensure that any tables are editable and in Word format, and wherever possible, embedded in the article file itself.

- Please ensure that the Article File you upload is a Word file.

- Please include an Abstract Figure file and an Abstract Figure legend. An appropriate figure legend, which should not exceed 150 words in length, should be included in the main manuscript file. The Abstract Figure is a piece of artwork

designed to give readers an immediate understanding of the research and should summarise the main conclusions. If possible, the image should be easily 'readable' from left to right or top to bottom. It should show the physiological relevance of the manuscript so readers can assess the importance and content of its findings. Abstract Figures should not merely recapitulate other figures in the manuscript. Please try to keep the diagram as simple as possible and without superfluous information that may distract from the main conclusion(s). Abstract Figures must be provided by authors no later than the revised manuscript stage and should be uploaded as a separate file during online submission labelled as File Type 'Abstract Figure'. Please also ensure that you include the figure legend in the main article file. All Abstract Figures should be created using BioRender. Authors should use The Journal's premium BioRender account to export high-resolution images. Details on how to use and access the premium account are included as part of this email.

----------------

EDITOR COMMENTS

Reviewing Editor:

The manuscript provides interesting insights into ultrasound-derived hemodynamic force (HDF) metrics in a murine TAC/deTAC model. However, while the study is technically well executed and the topic is timely, substantial conceptual, methodological, and interpretative concerns must be addressed before the work can be considered.

The analytical approach appears largely exploratory. A considerable number of HDF-derived variables are introduced and subsequently tested for statistical significance, without sufficient physiological justification for their selection. The relationship between HDF and established Doppler-derived intraventricular pressure gradients is not convincingly addressed, and the incremental mechanistic insight provided by HDF remains uncertain. In addition, given the small sample size, the robustness and generalizability of the statistical findings require more careful treatment, and predictive claims should be framed more cautiously.

Overall, the study has clear potential, but major revision is required before it can be reconsidered for publication.

Please use precise p values (no < or >) in figure 2.

-----------------

REFEREE COMMENTS

Referee #1:

This manuscript presents a pre-clinical study using echocardiography aiming to (1) compare conventional metrics (EF, global strain) with novel ones (flow-related hemodynamic forces) for description of LV function, and (2) verify the ability of these metrics to predict functional recovery after intervention. This is performed using a model of aortic constriction (banding) followed by de-banding.

The study is carefully performed by a methodological standpoint; results are interesting and original. They confirm the reliability of HDF-metrics to quantify LV function, like conventional indices, and identify specific HDF-metrics that appear to contain predictive potential, not contained in conventional indices.

The manuscript is well written and conceptually interesting. I appreciated the point about the need of mechanistically meaningful markers, and the aim to identify predictive methods-metrics.

I have only a few minor comments that can avoid confusion and may help improving the manuscript.

1. Please define TAC/deTAC in the introduction, before material and methods.

2. Substitute "intermittent" with "intermediate" in sentence "...speckle-tracking used to calculate intermittent boundary traces."

3. The slope of the HDF trace is called "acceleration" and "deceleration"; this can be misleading because the force itself is an acceleration (per unit mass). A reader may confuse the slope of the force, with an acceleration of the fluid/tissue that is not. I encourage to use a more physically-meaningful term, like "rate" (positive, early-systolic force-rate; negative late-systolic force-rate), or "slope" (systolic force up-slope; down-slope) or another.

4. The main result is about the HDF-metrics that significantly correlate with reverse LV functional recovery. The significance of the transverse force suggests a misalignment, did author tested if there is a similar significance of the force ratio or angle? On the same point, it would be useful to include a sentence about the author perspective concerning physics and/or physiology associated with this observation.

Referee #2:

In the present study, Day et al. evaluate changes in left ventricular hemodynamic forces in a murine TAC/deTAC model using high-resolution ultrasound and speckle-tracking analysis. The authors reconstruct longitudinal and transverse HDF components over the cardiac cycle, define several systolic and diastolic sub-intervals, and propose novel derived metrics-most notably a systolic acceleration-to-relaxation ratio-which they suggest are sensitive markers of dysfunction and recovery.

The manuscript is generally well written, the presentation is clear, and the figures are of high technical quality and effectively illustrate the proposed analysis framework. However, while the experimental model is well established and the imaging data appear technically adequate, I have major concerns regarding the conceptual framing of the study, the hypothesis-free nature of the analysis, and the interpretation of the HDF metrics.

General Comments:

1. Lack of clearly defined objectives and hypotheses: I found the scope and aim of the study not sufficiently well defined. The manuscript shows that it is possible to compute HDF metrics from murine echocardiography, but it is unclear what specific physiological or mechanistic question the study is designed to address.

A very large number of HDF-derived variables are introduced (global, systolic, diastolic, longitudinal, transverse, RMS values, normalized timings, ratios, angles), yet the authors do not specify a priori which of these metrics are hypothesized to change with pressure overload or recovery, nor why. As a result, the work reads largely as an exploratory analysis rather than a hypothesis-driven investigation.

2. Exploratory analysis strategy: Related to the above, the study gives the strong impression of a post hoc correlation-driven approach, in which many variables are computed and subsequently screened for statistical significance. In particular:

• The choice of derived metrics (e.g. acceleration-to-relaxation ratios, specific systolic sub-intervals) is not motivated by clear physiological reasoning.

• PCA and correlation analyses are applied after extensive metric generation, increasing the risk of over-interpretation, especially given the small sample size.

• The manuscript emphasizes statistically significant findings without adequately addressing the multiple-testing burden or the strong interdependence among many variables.

A more focused, hypothesis-driven analysis with a limited set of physiologically motivated metrics would substantially strengthen the scientific rigor of the work.

3. Relationship between HDF and Doppler-derived intraventricular pressure gradients: A central conceptual issue that is not addressed is to what extent HDF provide information beyond classical intraventricular pressure gradients (IVPGs) derived by integrating the Euler equation from Doppler velocity data. Both approaches are grounded in the same momentum balance governing blood acceleration. Doppler-derived IVPGs provide local, directional estimates of pressure gradients, whereas HDF represent volume-integrated pressure gradients expressed as vector quantities. However, the manuscript does not clarify:

• What hemodynamic information is uniquely captured by HDF that cannot, in principle, be obtained from Euler-based pressure gradient analysis.

• Whether the apparent advantages of HDF arise from genuine physiological insight or simply from spatial and temporal averaging.

• Why the proposed systolic and transverse HDF metrics should be expected to outperform established pressure-gradient-based measures.

Without a clear conceptual comparison, it remains uncertain whether HDF represent a meaningful advancement or primarily an alternative representation of similar information.

4. Incremental value relative to existing pressure-overload studies: The manuscript does not sufficiently position the work within the broader literature on aortic banding and pressure-overload models. Specifically, it remains unclear whether intraventricular pressure gradients or force-based metrics have been previously applied in TAC or similar models (using Doppler, MRI, or other techniques). Also it could be of interest to address how the present results compare with prior observations on systolic-diastolic pressure gradients, flow redirection, or ventricular remodeling under chronic overload. As currently written, the manuscript does not convincingly articulate the incremental mechanistic understanding gained relative to existing approaches.

5. Statistical robustness and generalizability: With only eight animals undergoing TAC/deTAC, statistical power is inherently limited. Several reported correlations and group differences appear sensitive to individual data points. Claims regarding prediction of recovery or superiority over conventional indices should therefore be substantially tempered and framed as exploratory.

Importantly, as measurements are repeated within the same animals across baseline, TAC, and deTAC conditions, treating observations as independent violates model assumptions. A linear mixed-effects model with each animal as a random effect would properly account for within-subject correlation and provide more reliable estimates of condition effects given the small sample size.

---

END OF COMMENTS

EDITOR COMMENTS

Reviewing Editor:

The manuscript provides interesting insights into ultrasound-derived hemodynamic force (HDF) metrics in a murine TAC/deTAC model. However, while the study is technically well executed and the topic is timely, substantial conceptual, methodological, and interpretative concerns must be addressed before the work can be considered.

The analytical approach appears largely exploratory. A considerable number of HDF-derived variables are introduced and subsequently tested for statistical significance, without sufficient physiological justification for their selection. The relationship between HDF and established Doppler-derived intraventricular pressure gradients is not convincingly addressed, and the incremental mechanistic insight provided by HDF remains uncertain. In addition, given the small sample size, the robustness and generalizability of the statistical findings require more careful treatment, and predictive claims should be framed more cautiously.

Overall, the study has clear potential, but major revision is required before it can be reconsidered for publication.

Please use precise p values (no < or >) in figure 2.

Thank you for the opportunity to revise this manuscript based on the reviewer comments and your suggestions. Figure 2 has been changed to reflect this recommendation as well as Table 1 and several supplemental tables.

REFEREE COMMENTS

Referee #1:

This manuscript presents a pre-clinical study using echocardiography aiming to (1) compare conventional metrics (EF, global strain) with novel ones (flow-related hemodynamic forces) for description of LV function, and (2) verify the ability of these metrics to predict functional recovery after intervention. This is performed using a model of aortic constriction (banding) followed by de-banding.

The study is carefully performed by a methodological standpoint; results are interesting and original. They confirm the reliability of HDF-metrics to quantify LV function, like conventional indices, and identify specific HDF-metrics that appear to contain predictive potential, not contained in conventional indices.

The manuscript is well written and conceptually interesting. I appreciated the point about the need of mechanistically meaningful markers, and the aim to identify predictive methods-metrics.
We greatly appreciate the kind words and opportunity to improve our manuscript.

I have only a few minor comments that can avoid confusion and may help improving the manuscript.

1. Please define TAC/deTAC in the introduction, before material and methods.
Thank you for the suggestion. We have now defined TAC/DeTAC in the introduction on page 6, line 10-18:

"The present study asks whether high-resolution ultrasound-derived HDF serves as a sensitive, mechanistically meaningful marker of left ventricular adaptation and recovery in the murine TAC-deTAC model, a surgical protocol of aortic constriction and subsequent debanding. The TAC/deTAC model enables the evaluation of dynamic changes in ventricular mechanics during loss and recovery of LV function (Ghajar-Rahimi *et al*., 2025)"

2. Substitute "intermittent" with "intermediate" in sentence "...speckle-tracking used to calculate intermittent boundary traces."
We have modified this sentence accordingly on page 8, lines 1-3:

"Endocardial and myocardial boundaries were delineated at end-systole and end-diastole using the Vevo Strain software, with speckle-tracking used to calculate intermediate boundary traces."

3. The slope of the HDF trace is called "acceleration" and "deceleration"; this can be misleading because the force itself is an acceleration (per unit mass). A reader may confuse the slope of the force, with an acceleration of the fluid/tissue that is not. I encourage to use a more physically-

meaningful term, like "rate" (positive, early-systolic force-rate; negative late-systolic force-rate), or "slope" (systolic force up-slope; down-slope) or another.

Thank you for the suggestion. We agree that the original terminology was misleading as the terms acceleration and relaxation were representing the root mean squared longitudinal force term over specific intervals and not rate or slope terms. To better describe these intervals for the reader, the term "systolic acceleration" has been changed to "early systolic impulse", the term "systolic relaxation" has been changed to "late systolic impulse", and "systolic acceleration to relaxation ratio" has been changed accordingly to "early-to-late systolic impulse longitudinal force ratio". Figure 2 Panel F and G, Figure 3 Panel A, Supplemental figure 1, Table 1, and Supplemental Table 2 have all been adjusted to match the modified metric names. The following text has also been adjusted to match the terminology on page 3 line 14:

"Notably, a novel early-to-late systolic impulse longitudinal HDF ratio showed the strongest associations with pressure overload and subsequent functional recovery (baseline vs TAC: $p < 0.001$; TAC vs deTAC: $p < 0.001$; baseline vs deTAC: $p = 0.043$)."

Page 9 line 3 and line 5:

> **"Early Systolic Impulse:** The interval from the HDF zero-crossing (or systolic minimum) immediately preceding the systolic peak, up to the systolic peak."
>
> **"Late Systolic Impulse:** The interval from the systolic peak to the end of the systolic impulse (the zero-crossing immediately preceding end-systole)."

Page 12, line 3-6:
"Among these parameters, the early systolic impulse RMS of longitudinal HDF was significantly reduced in TAC hearts compared to both baseline and deTAC hearts ($p$<0.01 and $p$<0.001, Figure 2F)). Interestingly, the early-to-late systolic impulse longitudinal force ratio was significantly increased during TAC compared to baseline and deTAC ($p$<0.001, Figure 2G)."

Page 12, line 18:
"Most HDF metrics show a positive correlation with LV ejection fraction and aortic peak velocity, except for the e-wave longitudinal RMS, which behaves unpredictably, and the early-to-late systolic impulse longitudinal force ratio, which shows a predictable but inverse relationship (Figure 3A)."

4. The main result is about the HDF-metrics that significantly correlate with reverse LV functional recovery. The significance of the transverse force suggests a misalignment, did author tested if there is a similar significance of the force ratio or angle? On the same point, it would be useful to include a sentence about the author perspective concerning physics and/or physiology associated with this observation.

We thank the reviewer for this comment. We tested the significance of both the force ratios and angle for the same time intervals that transverse and longitudinal components were tested. The results of these tests are summarized in Table 1, where none of the metrics in question were found to be significant. We have also added to the discussion the additional mention of HDF angle along with e-wave in discussion of significant vs. non-significant metrics and an additional section in the results section on page 12 line 23-26, and page 15 line 6 and 15:

"HDF angle was tested at systolic and diastolic intervals, and despite what previous studies have found in humans, HDF angle was non-significant in all cases (Table 1)."
"In this TAC and deTAC model, systolic impulse metrics were generally found to have much higher significance as compared to the diastolic e-wave and HDF angle metrics."
"Building on this, PCA of combined HDF and conventional echocardiographic variables revealed that systolic HDF features contributed most strongly to the main axis separating baseline, TAC, and deTAC conditions, whereas diastolic e-wave and HDF angle related metrics had only modest influence on group discrimination."

Referee #2:

In the present study, Day et al. evaluate changes in left ventricular hemodynamic forces in a murine TAC/deTAC model using high-resolution ultrasound and speckle-tracking analysis. The authors reconstruct longitudinal and transverse HDF components over the cardiac cycle, define several systolic and diastolic sub-intervals, and propose novel derived metrics-most notably a systolic acceleration-to-relaxation ratio-which they suggest are sensitive markers of dysfunction and recovery.

The manuscript is generally well written, the presentation is clear, and the figures are of high technical quality and effectively illustrate the proposed analysis framework. However, while the experimental model is well established and the imaging data appear technically adequate, I have major concerns regarding the conceptual framing of the study, the hypothesis-free nature of the analysis, and the interpretation of the HDF metrics.

We are very appreciative of the supportive words and opportunity to improve this manuscript.

General Comments:

1. Lack of clearly defined objectives and hypotheses: I found the scope and aim of the study not sufficiently well defined. The manuscript shows that it is possible to compute HDF metrics from murine echocardiography, but it is unclear what specific physiological or mechanistic question the study is designed to address.

A very large number of HDF-derived variables are introduced (global, systolic, diastolic, longitudinal, transverse, RMS values, normalized timings, ratios, angles), yet the authors do not specify a priori which of these metrics are hypothesized to change with pressure overload or recovery, nor why. As a result, the work reads largely as an exploratory analysis rather than a hypothesis-driven investigation.

We thank the reviewer for this helpful comment. We have revised the Introduction to clarify that the specific physiological question addressed by this study is whether ultrasound-derived HDF can sensitively capture the mechanical consequences of pressure overload and unloading in the

mouse left ventricle, and whether interval-specific systolic HDF metrics can predict reverse remodeling after debanding better than conventional indices such as EF and strain. We also clarified that the mechanistic focus is on intraventricular force generation and redirection during systole as a marker of maladaptive remodeling and recovery in the TAC/deTAC model.

We have added the following to the end of the introduction to align with this suggestion on page 6:
"We ask in the present study whether high-resolution ultrasound-derived HDF serves as a sensitive, mechanistically meaningful marker of left ventricular adaptation and recovery in the murine TAC-deTAC model, a surgical protocol of aortic constriction and subsequent debanding. The TAC/deTAC model enables the evaluation of dynamic changes in ventricular mechanics during loss and recovery of LV function (Ghajar-Rahimi *et al*., 2025). Given the inherent complexity of HDF waveforms and their derived parameters based on intraventricular pressure gradients, a central aim of this work was to identify HDF metrics that are most representative of cardiac functional status and to establish robust quantification strategies for their extraction in a preclinical ultrasound setting. Specifically, we tested whether interval-specific systolic HDF metrics capture changes in intraventricular force generation and flow redirection induced by TAC, and whether these parameters better predict functional recovery after deTAC than conventional echocardiographic indices."

2. Exploratory analysis strategy: Related to the above, the study gives the strong impression of a post hoc correlation-driven approach, in which many variables are computed and subsequently screened for statistical significance. In particular:

• The choice of derived metrics (e.g. acceleration-to-relaxation ratios, specific systolic sub-intervals) is not motivated by clear physiological reasoning.

We thank the reviewer for this comment. We agree that our original text did not sufficiently explain the physiological basis for selecting specific HDF-derived parameters. Our approach was not purely post-hoc, but we were guided by prior clinical and preclinical literature on HDF and by the known biomechanics of pressure overload (see references in table below).

In particular, systolic impulse and its sub-intervals were selected because they reflect the phase of active force generation during LV ejection under increased afterload. The systolic impulse interval captures the rise in forward force production, whereas the systolic relaxation interval reflects deceleration of force generation as systole progresses toward aortic valve closure. The acceleration-to-relaxation ratio was therefore introduced as a composite index of systolic force balance and efficiency.

We also examined transverse systolic impulse because transverse force components may reflect altered flow organization and non-axial force redirection in the remodeling ventricle. We have now revised the manuscript to better justify these metrics and to clarify that this work is intended as an exploratory, hypothesis-generating analysis focused on identifying the HDF descriptors most sensitive to pressure overload and reverse remodeling.

We modified the manuscript with the following addition to reflect the initial oversight of not including physiological reasoning for the defined HDF descriptors on page 8 lines 15-22:

"The selected HDF descriptors were chosen as *priori* based on published findings and their physiological interpretation (Arvidsson *et al.*, 2018; Backhaus *et al.,* 2022; Cesareo *et al.*, 2024; Daal *et al.,* 2022; Dal Ferro *et al.,* 2019; Eriksson *et al.*, 2016, 2017; Faganello *et al.,* 2024; Li *et al.*, 2025; Pedrizzetti *et al.,* 2016, 2017; Pola *et al.,* 2022, 2023; Sjöberg *et al.,* 2018; Vairo *et al.,* 2023). In particular, the systolic impulse based metrics reflect the active force generation during LV ejection under increased afterload and the diastolic E-wave reflects desynchrony in left atrium and ventricle during early filling of the LV. We define the following specific metrics to maintain consistent data collection across samples:"

Further, we have provided a summary table from the literature in this response document to show what informed our decisions for interval selection. We also propose, however, that utilizing non-heuristic approaches as well as basing metrics on previous publications improves the overall characterization of the HDF metric.

| **Study Population** | **Imaging Modality** | **Parameters of Importance** | **Findings (summarized from abstract)** | **Reference** |
|---|---|---|---|---|
| LBBB | CMR (4D flow MRI) | E-wave transverse; longitudinal RMS; Diastole Longitudinal Force | Patients with LBBB exhibited a loss of longitudinal forces in diastole and increased transverse force magnitudes compared to controls. Filling forces were more orthogonal to the main LV flow direction, and greater conduction abnormalities correlated with higher discordance between filling force and flow direction. | Eriksson J, Zajac J, Alehagen U, Bolger AF, Ebbers T, Carlhäll CJ. Left ventricular hemodynamic forces as a marker of mechanical dyssynchrony in heart failure patients with left bundle branch block. Sci Rep. 2017 Jun 7;7(1):2971. doi: 10.1038/s41598-017-03089-x. PMID: 28592851; PMCID: PMC5462838. |
| LBBB with CRT Treatment | CMR (4D flow MRI) | Systolic Peak | Non-responders to CRT had smaller HDF in the inferior-anterior direction (systole) and apex- | Pola K, Roijer A, Borgquist R, Ostenfeld E, Carlsson M, Bakos Z, Arheden H, Arvidsson PM. Hemodynamic forces from 4D flow magnetic resonance imaging predict |

| | | | | |
|---|---|---|---|---|
| | | | base direction (diastole). A larger diastolic HDF ratio (transverse/longitudinal) was identified as a significant predictor for identifying non-responders to CRT. | left ventricular remodeling following cardiac resynchronization therapy. J Cardiovasc Magn Reson. 2023 Aug 25;25(1):45. doi: 10.1186/s12968-023-00955-8. PMID: 37620886; PMCID: PMC10463519. |
| HFpEF in mice (doxycycline vs control) | CMR | overall (not RMS) transverse force; Longitudinal RMS | In a db/db mouse model of HFpEF, hemodynamic force RMS values, average forces, and transverse/longitudinal ratios were significantly lower compared to controls. Hemodynamic force parameters were the only functional outcomes showing distinct temporal differences between groups as the disease progressed. | Daal, M.R.R., Strijkers, G.J., Hautemann, D.J. et al. Longitudinal CMR assessment of cardiac global longitudinal strain and hemodynamic forces in a mouse model of heart failure. Int J Cardiovasc Imaging 38, 2385–2394 (2022). https://doi.org/10.1007/s10554-022-02631-x |
| Fabry Disease w/ and w/o LV Hypertrophy | CMR | Systolic Impulse; Systolic Peak Force | Patients with Fabry disease showed increased systolic impulse and systolic peak forces compared to healthy controls. Notably, the systolic impulse increased even in the pre-hypertrophic stage and correlated positively with disease severity, offering potential for early diagnosis. | Li J, Liang S, Xu Z, Wan K, Pu L, Wang J, Han Y, Chen Y. Left Ventricular Hemodynamic Forces Changes in Fabry Disease: A Cardiac Magnetic Resonance Study. J Magn Reson Imaging. 2025 Jan 22. doi: 10.1002/jmri.29700. Epub ahead of print. PMID: 39843855. |

| | | | | |
|---|---|---|---|---|
| CRT Treated Individuals | Echo (Speckle Tracking) | RMS transvere; Overall Longitudinal to Transverse Ratio | Following CRT, pacing-induced realignment of HDFs led to a significant reduction in the ratio between transverse and longitudinal force amplitudes. This restoration of the normal force orientation was observed independently of immediate changes in ejection fraction and strain metrics. | M. Dal Ferro et al., “Left Ventricular Response to Cardiac Resynchronization Therapy: Insights From Hemodynamic Forces Computed by Speckle Tracking,” Frontiers in Cardiovascular Medicine, vol. 6, May 2019, doi: https://doi.org/10.3389/fcvm.2019.00059. |
| CRT Treatment | CMR (4D flow MRI) | RMS transverse; longitudinal ratio overall; paired longitudinal force between systole and diastole | Heart failure patients with LV dyssynchrony exhibited altered hemodynamic forces, including a loss of longitudinal diastolic forces and increased transverse force magnitudes. The diastolic force ratio successfully differentiated healthy from diseased ventricles independently of traditional dyssynchrony measures. | P. M. Arvidsson et al., “Hemodynamic forces using four-dimensional flow MRI: an independent biomarker of cardiac function in heart failure with left ventricular dyssynchrony?,” AJP Heart and Circulatory Physiology, vol. 315, no. 6, pp. H1627–H1639, Sep. 2018, doi: https://doi.org/10.1152/ajpheart.00112.2018. |
| Pulmonary Hypertension Precapillary | CMR (4D flow MRI) | Late diastolic longitudinal | In precapillary pulmonary hypertension, biventricular HDF (indexed to stroke volume) were elevated in all spatial directions. The right ventricle | Pola K, Bergström E, Töger J, Rådegran G, Arvidsson PM, Carlsson M, Arheden H, Ostenfeld E. Increased biventricular hemodynamic forces in precapillary pulmonary hypertension. Sci Rep. 2022 Nov 19;12(1):19933. doi: 10.1038/s41598-022-24267-6. |

| | | | | |
|---|---|---|---|---|
| | | | compensates for increased afterload by augmenting transverse forces, while left ventricular hemodynamic abnormalities primarily result from underfilling. | PMID: 36402861; PMCID: PMC9675772. |
| repaired TOF with Pulmonary valve Regurgitation before and after valve replacement | CMR (4D flow MRI) | RMS transverse ; Longitudinal ratio | Patients with repaired tetralogy of Fallot and right ventricular volume overload due to pulmonary regurgitation exhibit significantly altered biventricular hemodynamic forces. These alterations reflect impaired pumping efficiency and abnormal flow routing within the ventricles. | Sjöberg P, Töger J, Hedström E, Arvidsson P, Heiberg E, Arheden H, Gustafsson R, Nozohoor S, Carlsson M. Altered biventricular hemodynamic forces in patients with repaired tetralogy of Fallot and right ventricular volume overload because of pulmonary regurgitation. Am J Physiol Heart Circ Physiol. 2018 Dec 1;315(6):H1691-H1702. doi: 10.1152/ajpheart.00330.2018. Epub 2018 Sep 28. PMID: 30265559. |
| Dilated Cardiomyopathy | CMR (4D flow MRI) | Transverse max; Longitudinal max in LV during E-wave diastole | Patients with dilated cardiomyopathy display significantly altered left ventricular hemodynamic forces compared to healthy subjects. These force profiles reflect an abnormal coupling between blood flow momentum and the underlying myocardial wall mechanics. | Eriksson J, Bolger AF, Ebbers T, Carlhäll CJ. Assessment of left ventricular hemodynamic forces in healthy subjects and patients with dilated cardiomyopathy using 4D flow MRI. Physiol Rep. 2016 Feb;4(3):e12685. doi: 10.14814/phy2.12685. PMID: 26841965; PMCID: PMC4758930. |
| Non-ischemic dilated | Echo | HDF Angle in first | Echocardiography-derived LV hemodynamic | Cesareo M, Ródenas-Alesina E, Guala A, Lozano-Torres J, Casas G, Vallelonga F, Airale L, Ferreira- |

| | | | | |
|---|---|---|---|---|
| cardiomyopathy predicting major adverse cardiovascular events (MACE) | | 2/3rds of systole vs last 1/3rd of systole | forces provide significant prognostic value in patients with non-ischemic dilated cardiomyopathy. Altered force profiles and abnormal flow momentum distributions are associated with worse long-term clinical outcomes. | González I, Milan A, Rodriguez-Palomares JF. Echocardiography-Derived Hemodynamic Forces Are Associated with Clinical Outcomes in Patients with Non-Ischemic Dilated Cardiomyopathy. J Clin Med. 2024 Jun 30;13(13):3862. doi: 10.3390/jcm13133862. PMID: 38999432; PMCID: PMC11242817. |
| HFpEF in humans | CMR | RMS E-wave apical-basal early diastolic filling | In patients with heart failure with preserved ejection fraction (HFpEF), hemodynamic forces differ significantly from controls. HDF analysis provided unique insights into impaired diastolic filling mechanics and highlighted early functional deterioration despite preserved ejection fractions. | Backhaus SJ, Uzun H, Rösel SF, Schulz A, Lange T, Crawley RJ, Evertz R, Hasenfuß G, Schuster A. Hemodynamic force assessment by cardiovascular magnetic resonance in HFpEF: A case-control substudy from the HFpEF stress trial. EBioMedicine. 2022 Dec;86:104334. doi: 10.1016/j.ebiom.2022.104334. Epub 2022 Nov 21. PMID: 36423376; PMCID: PMC9691873. |
| aortic coarctation with preserved LV systolic function | Echo | Total LV Impulse | Adult patients with treated aortic coarctation and preserved systolic function demonstrate altered left ventricular hemodynamic forces compared to controls. These changes reflect subtle, subclinical alterations in mechanical function and blood- | Faganello G, Collia D, Pagura L, Croatto E, Tosoni LM, Toritto P, Pedrizzetti G, Lenarda AD. Impact of left ventricular hemodynamic forces in adult patients with treated aortic coarctation and preserved left ventricular systolic function. Echocardiography. 2024 Jan;41(1):e15742. doi: 10.1111/echo.15742. PMID: 38284680. |

| | | | | |
|---|---|---|---|---|
| | | | myocardium interactions. | |
| Severe Aortic Stenosis (Undergoing TAVI) | Echo | LV Systolic Longitudinal Force; LV Impulse | Transcatheter Aortic Valve Implantation (TAVI) leads to an acute modification of hemodynamic forces in patients with severe aortic stenosis. Relief of pressure overload yields rapid improvements in force alignment and intraventricular pressure gradients. | Vairo A, Zaccaro L, Ballatore A, Airale L, D'Ascenzo F, Alunni G, Conrotto F, Scudeler L, Mascaretti D, Miccoli D, Torre M La, Rinaldi M, Pedrizzetti G, Salizzoni S & De Ferrari GM (2023). Acute Modification of Hemodynamic Forces in Patients with Severe Aortic Stenosis after Transcatheter Aortic Valve Implantation. J Clin Med; DOI: 10.3390/JCM12031218/S1. |
| Heterogeneous subjects (Validation study vs 4D flow MRI) | CMR & Echo (Validation) | Longitudinal force at Systole-Diastole transition period | A simplified model based on endocardial tissue dynamics reliably estimates intraventricular pressure gradients and global HDF vector features without requiring internal velocity field data. The method showed high concordance when validated against 4D flow MRI measurements. | Pedrizzetti G, Arvidsson PM, Töger J, Borgquist R, Domenichini F, Arheden H & Heiberg E (2017). On estimating intraventricular hemodynamic forces from endocardial dynamics: A comparative study with 4D flow MRI. J Biomech 60, 203–210. |
| Heart Failure (Undergoing CRT) | Echo (PIV) | HDF Angle in Systole and Diastole; HDF Angle change after CRT therapy | Discontinuation of CRT pacing causes an immediate deviation of blood flow momentum from its normal base-apex orientation. The degree of re-alignment of these hemodynamic forces correlates | Pedrizzetti G, Martiniello AR, Bianchi V, D'Onofrio A, Caso P, Tonti G. Changes in electrical activation modify the orientation of left ventricular flow momentum: novel observations using echocardiographic particle image velocimetry. Eur Heart J Cardiovasc Imaging. 2016 Feb;17(2):203-9. doi: 10.1093/ehjci/jev137. Epub 2015 Jun 9. PMID: 26060201; PMCID: PMC4882880. |

| | | | | |
|---|---|---|---|---|
| | | | significantly with long-term volumetric reduction and positive CRT outcomes. | |

• PCA and correlation analyses are applied after extensive metric generation, increasing the risk of over-interpretation, especially given the small sample size.

We thank the reviewer for this important comment. We agree that, in the context of a small cohort and a large number of derived variables, correlation and PCA analyses may increase the risk of over-interpretation if presented as confirmatory tests. Our intention was not to claim independent validation of a predictive model, but rather to use these analyses as exploratory methods to identify the HDF features that best separate conditions and appear most closely linked to ventricular remodeling in this TAC/deTAC setting.

To address this concern, we have revised the text to make clear that these multivariate analyses are hypothesis-generating and should be interpreted cautiously given the sample size and the number of tested parameters. We now emphasize that the PCA was used to summarize overall structure in the dataset, while the correlation analyses were used to identify candidate associations for future validation, rather than to establish definitive causal or predictive relationships. We have included the following in the limitations section on page 16 lines 10-12 to address these concerns:

"As a result of the small study cohort (n=8), these multivariate analyses are mainly hypothesis-generating and are not necessarily reflective of a causative relationship. Instead, our PCA analysis was used to summarize overall structure of our dataset and help identify correlative relationships."

• The manuscript emphasizes statistically significant findings without adequately addressing the multiple-testing burden or the strong interdependence among many variables.

A more focused, hypothesis-driven analysis with a limited set of physiologically motivated metrics would substantially strengthen the scientific rigor of the work.

We thank the reviewer for this insight. This comment was an overarching ethos as we revised the manuscript for all comments that were received. We have compiled these changes below with where they can be found.

The study not explicitly asks if HDF can sensitively capture mechanical consequences of pressure overload and identifies our predictive hypothesis and central aim on page 6 line 10-14 and line 22-25:

“We ask in the present study whether high-resolution ultrasound-derived HDF serves as a sensitive, mechanistically meaningful marker of left ventricular adaptation and recovery in the murine TAC-deTAC model, a surgical protocol of aortic construction and subsequent debanding.”
“Specifically, we tested whether interval-specific systolic HDF metrics capture changes in intraventricular force generation and flow redirection induced by TAC, and whether these parameters better predict functional recovery after deTAC than conventional echocardiographic indices.”

To align with identifying a limited set of physiologically motivated metrics, we have included the following to the methods section on page 8 line 15-22 and the summary table of candidate markers in this document on pages 5-11:

“The selected HDF descriptors were chosen as *priori* based on published findings and their physiological interpretation (Arvidsson *et al.*, 2018; Backhaus *et al.,* 2022; Cesareo *et al.*, 2024; Daal *et al.,* 2022; Dal Ferro *et al.,* 2019; Eriksson *et al*., 2016, 2017; Faganello *et al.,* 2024; Li *et al.*, 2025; Pedrizzetti *et al.,* 2016, 2017; Pola *et al.,* 2022, 2023; Sjöberg *et al.,* 2018; Vairo *et al.,* 2023). In particular, the systolic impulse based metrics reflect the active force generation during LV ejection under increased afterload and the diastolic E-wave reflects desynchrony in left atrium and ventricle during early filling of the LV. We define the following specific metrics to maintain consistent data collection across samples:”

To address the multiple-test burden we now define frame PCA as a hypothesis generating exploratory method rather than a predictive model in the limitation on page 16 line 10-12:

“As a result of the small study cohort (n=8), these multivariate analyses are mainly hypothesis-generating and are not necessarily reflective of a causative relationship. Instead, our PCA analysis was used to summarize overall structure of our dataset and help identify correlative relationships.”

3. Relationship between HDF and Doppler-derived intraventricular pressure gradients: A central conceptual issue that is not addressed is to what extent HDF provide information beyond classical intraventricular pressure gradients (IVPGs) derived by integrating the Euler equation from Doppler velocity data. Both approaches are grounded in the same momentum balance governing blood acceleration. Doppler-derived IVPGs provide local, directional estimates of pressure gradients, whereas HDF represent volume-integrated pressure gradients expressed as vector quantities. However, the manuscript does not clarify:

• What hemodynamic information is uniquely captured by HDF that cannot, in principle, be obtained from Euler-based pressure gradient analysis. Whether the apparent advantages of HDF arise from genuine physiological insight or simply from spatial and temporal averaging.
• Why the proposed systolic and transverse HDF metrics should be expected to outperform established pressure-gradient-based measures.

Without a clear conceptual comparison, it remains uncertain whether HDF represent a meaningful advancement or primarily an alternative representation of similar information.

We appreciate the insightful query regarding the conceptual and physical distinction between Hemodynamic Forces (HDF) and Intraventricular Pressure Gradients (IVPG). While both are grounded in the same Euler momentum balance, they represent different scales of cardiac interaction. While the Euler-based pressure gradient analysis can provide 1D IVPGs, the HDF volumetrically averages the entire ventricle and can be used to determine if the IVPG aligns with the outflow tract or if it is dissipated through the transverse pressure against the ventricular walls. In other words, HDF quantifies the efficiency of energy usage and IVPG production by the LV through the comparison of transverse and longitudinal components. It also has the advantage of calculating a true vector as opposed to a scalar value from color Doppler such that you can concurrently compare atrial driven ventricular filling to myocardium driven ventricular emptying. To get the same information from Euler derived IVPGs, the adjustment of color Doppler framing may be physiologically inaccurate as it would require comparisons over different cardiac cycles.

We show here that HDF metrics outperform 1D pressure-gradient measures specifically in a pressure-overload TAC model because they capture the consequences of spherical remodeling. In agreement with previous reports, pressure overload causes the ventricle to become more spherical due to eccentric remodeling (Opie L, Commerford P, Gersh B et al. *Controversies in ventricular remodelling.* The Lancet, 367, 356-367). While IVPG would potentially show consistent IVPG in the longitudinal direction (measured at the outflow tract), HDF can also identify changes in transverse IVPG components. Still, we were limited in quantifying this actual impact due to the 2D nature of our calculations, something that could be addressed with a 3D approach. Because HDF is a whole volume integration of the IVPG, HDF is less sensitive to local noise or the specific placement of the Doppler sample volume, acting like a global filter (Eriksson *et al.*, 2017; Vallelonga *et al.*, 2021). While further work with additional disease models and patient images is needed, the data included here suggest that HDF is likely a better metric for quantifying reductions in ventricular efficiency compared to IVPG alone. We have revised the limitations to explicitly address these differences as follows on page 16 lines 6-9:

"The use of HDF metrics show theoretical benefits over Euler IVPG, but we did not assess eccentric remodeling (Opie *et al.,* 2006) so a direct benefit over traditional Euler IVPGs is not explored in this study"

4. Incremental value relative to existing pressure-overload studies: The manuscript does not sufficiently position the work within the broader literature on aortic banding and pressure-overload models. Specifically, it remains unclear whether intraventricular pressure gradients or force-based metrics have been previously applied in TAC or similar models (using Doppler, MRI, or other techniques). Also it could be of interest to address how the present results compare with prior observations on systolic-diastolic pressure gradients, flow redirection, or ventricular

remodeling under chronic overload. As currently written, the manuscript does not convincingly articulate the incremental mechanistic understanding gained relative to existing approaches.

We thank the reviewer for raising the important point about positioning this work within the broader literature on aortic banding and pressure overload models. As far as we have seen, there is one experimental study that exists testing HDF in mice which uses CMR (Daal *et al*., 2022). Beyond this work, all research has been done in human subjects, indicating a significantly limited scope in terms of idealized and uniform overload-induced adaption cases to analyze using the HDF metrics. The work that has been explored in human models is primarily done in CMR (Arvidsson *et al*., 2018; Backhaus *et al.,* 2022; Eriksson *et al*., 2016, 2017; Li *et al.*, 2025; Pedrizzetti *et al.,* 2017; Pola *et al.,* 2022, 2023; Sjöberg *et al.,* 2018). We position this work such that we build on the verification of the HDF metrics from Pedrizzetti *et al*. 2017 across Echo and CMR and try to determine the validity of HDF metrics in a murine model, a model that was first shown with CMR in Daal *et al*. 2022. We position this work to expand previous findings in human models using ultrasound (Cesareo *et al.*, 2024; Dal Ferro *et al.,* 2019; Faganello *et al.,* 2024; Pedrizzetti *et al.,* 2016, 2017; Vairo *et al.,* 2023).

We have added the following to the introduction section on page 5-6 line 29-30 and 1-2 to include a crystalized form of this response:

This work is thus positioned to expand the findings of human research using ultrasound (Cesareo *et al.*, 2024; Dal Ferro *et al.,* 2019; Faganello *et al.,* 2024; Pedrizzetti *et al.,* 2016, 2017; Vairo *et al.,* 2023) to a murine model that has been previously investigated using CMR (Daal *et al.*, 2022).

5. Statistical robustness and generalizability: With only eight animals undergoing TAC/deTAC, statistical power is inherently limited. Several reported correlations and group differences appear sensitive to individual data points. Claims regarding prediction of recovery or superiority over conventional indices should therefore be substantially tempered and framed as exploratory.

We thank the reviewer for this important comment. We agree that the modest sample size could limit statistical power and generalizability, meaning that several correlations may have been influenced by individual variability. We have therefore revised the manuscript to present these findings as more exploratory and hypothesis-gathering rather than definitive. In particular, we have softened claims regarding prediction of recovery and superiority over conventional indices, and we now emphasize that the present study establishes feasibility and identifies candidate HDF markers that warrant further investigation in larger cohorts. We made the following modifications to soften these claims.

The key points have been modified on page 4 line 7-8:

"Systolic HDF metrics, particularly transverse components during impulse intervals showed potential for predictive power correlating with cardiac recovery."

The results section title has been modified on page 12 line 10-11:

**"Interval-Specific Hemodynamic Forces Show Promise for Improved Correlative Tracking of Ventricular Remodeling Compared to Strain"**

The results section has also been modified on page 13 line 11 and 15:

"Finally, we assessed whether any HDF or conventional metrics measured during TAC showed associations with the reversal of LV functional impairment after debanding. Using linear mixed-effects correlation analysis (Supplemental Tables 2 and 3), we found that among the 41 parameters tested, only HDF-derived metrics, specifically the systolic transverse RMS and systolic transverse impulse, ~~significantly~~ correlate with reverse LV functional recovery (Figure 3C), whereas ejection fraction failed to show any predictive value (Figure 3D)."

The discussion has been modified on page 13 line 23:

"Beyond this concordance, our findings indicate that HDF metrics offer added value by identifying candidate markers of left ventricular remodeling and functional recovery following surgical intervention."

The conclusion has been modified on page 16 and lines 19-23:

"This preclinical study establishes ultrasound-derived HDF metrics, particularly systolic transverse impulse components, as possible predictors of LV functional recovery following aortic debanding in a mouse TAC model, establishing baseline performance in line with conventional strain and ejection fraction measures at single time points while laying the groundwork for further work to show potential advantages of the metric."

Importantly, as measurements are repeated within the same animals across baseline, TAC, and deTAC conditions, treating observations as independent violates model assumptions. A linear mixed-effects model with each animal as a random effect would properly account for within-subject correlation and provide more reliable estimates of condition effects given the small sample size.

We thank the reviewer for the important acknowledgement of a more statistically robust method of analyzing our results. We reran the statistics using a linear mixed-effects model (LMM). The methods section has been changed to align with the updated statistical methods and mentions of the statistical methods in discussion have been edited to align with the change on page 9:

"All statistical analysis was performed with R version 4.5.1 (RStudio), and the data are presented as mean ± standard deviation (SD). We used linear mixed-effects modeling (LMM) to compare baseline, 3-week TAC, and 4-week post-deTAC global parameters. For interval timing metrics analysis, we used both the raw and normalized data for each timing metric to the overall RR interval length. We also used LMM to describe relationships between 3-week TAC and 4-week post-deTAC variables."

Given the number of tests we ran, we also accounted for family wise error rate using a Benjamini-Hochberg step-up procedure to temper p values. To account for this change, following has been added to the methods section on page 9:

"Because HDF orientations represent directional data on a continuous 360-degree periodic scale, standard linear statistical methods are invalid. Consequently, all angular parameters were evaluated using dedicated circular statistics, adhering to the theoretical frameworks established by Mardia and Jupp (Mardia & Jupp, 1999). Changes in the mean direction of HDF vectors between independent groups were assessed using the Watson-Williams multi-sample test. To specifically account for the longitudinal, repeated-measures design of the TAC and deTAC interventions within the same subjects, the paired Watson-Williams test was utilized to evaluate intra-subject angular shifts over time. All circular parameters are reported as the circular mean direction ± circular standard deviation."

This line of rerunning statistics also highlighted an oversight in calculating HDF angle statistics which have now been rerun using circular statistics. To account for this change, following has been added to the methods section on page 10:

"To control the false discovery rate (FDR) associated with generating multiple exploratory Hemodynamic Force (HDF) parameters, p-values were adjusted using the Benjamini-Hochberg step-up procedure. To prevent mathematical over-penalization of inherently collinear variables, parameters were a priori divided into four distinct physiological families: Clinical Standards, Global HDF, Systolic HDF, and Diastolic HDF. The FDR correction was applied independently within each functional family across the three longitudinal time points (Baseline, TAC, deTAC). Furthermore, normalized interval metrics (e.g., Normalized Systole Length) were strictly treated as mathematically redundant equivalents of their raw counterparts during the rank-ordering phase of the FDR algorithm to preserve statistical power. An FDR-adjusted p-value of $p < 0.05$ was considered statistically significant."

Dear Dr Sicard,

Re: JP-RP-2026-290877R1 "**Ultrasound-Based Hemodynamic Force Trajectories in a Murine Model of Reversible Pressure Overload**" by Peyton Day, Thomas Moore-Morris, Craig J Goergen, and Pierre Sicard

Thank you for submitting your manuscript to The Journal of Physiology. It has been assessed by a Reviewing Editor and by 2 expert referees and we are pleased to tell you that it is acceptable for publication following satisfactory revision.

The referee reports are copied at the end of this email.

Please address all the points raised and incorporate all requested revisions or explain in your Response to Referees why a change has not been made. We hope you will find the comments helpful and that you will be able to return your revised manuscript within 4 weeks. If you require longer than this, please contact journal staff: jp@physoc.org.

Your revised manuscript should be submitted online using the link in your Author Tasks Link Not Available. This link is accessible via your account as Corresponding Author; it is not available to your co-authors. If this presents a problem, please contact journal staff (jp@physoc.org). Image files from the previous version are retained on the system. Please ensure you replace or remove any files that are being revised.

If you do not wish to submit a revised version of your manuscript, you must inform our journal staff (jp@physoc.org) or reply to this email to request withdrawal. Please note that a manuscript must be formally withdrawn from the peer review process at one journal before it may be submitted to another journal.

TRANSPARENT PEER REVIEW POLICY: To improve the transparency of its peer review process The Journal of Physiology publishes online, as supporting information, the peer review history of all articles accepted for publication. Readers will have access to decision letters, including Editors' comments and referee reports, for each version of the manuscript, as well as any author responses to peer review comments. Referees can decide whether or not they wish to be named on the peer review history document.

ABSTRACT FIGURES: Authors are expected to use The Journal's premium BioRender account to create/redraw their Abstract Figures. Information on how to access this account is here: https://physoc.onlinelibrary.wiley.com/journal/14697793/biorender-access.

This will enable Authors to create and download high-resolution figures. If authors have used the free BioRender service, they can use the instructions provided in the link above to download a high-resolution version suitable for publication.

The link provided should only be used for the purposes of this submission. Authors will be charged for figures created on this account if they are not related to this manuscript submission.

LANGUAGE EDITING AND SUPPORT FOR PUBLICATION: If you would like help with English language editing, or other article preparation support, Wiley Editing Services offers expert help, including English Language Editing, as well as translation, manuscript formatting, and figure formatting at www.wileyauthors.com/eeo/preparation. You can also find resources for Preparing Your Article for general guidance about writing and preparing your manuscript at www.wileyauthors.com/eeo/prepresources.

RIGOUR AND REPRODUCIBILITY: We recommend authors consult The Journal's Rigour and Reproducibility requirements to ensure that their manuscript is suitable for publication: https://physoc.onlinelibrary.wiley.com/pb-assets/hub-assets/physoc/documents/TJP-Rigour-and-Reproducibility-Requirements.pdf

REVISION CHECKLIST:

Check that your Methods section conforms to journal policy: https://jp.msubmit.net/cgi-bin/main.plex?form_type=display_requirements#methods

Check that data presented conforms to the statistics policy: https://jp.msubmit.net/cgi-bin/main.plex?form_type=display_requirements#statistics

Upload a full Response to Referees file. To create your 'Response to Referees' copy all the reports, including any comments from the Senior and Reviewing Editors, into a Microsoft Word, or similar, file and respond to each point, using font or background colour to distinguish comments and responses and upload as the required file type.

Please upload two versions of your manuscript text: one with all relevant changes highlighted and one clean version with no changes tracked. The manuscript file should include all tables and figure legends, but each figure/graph should be uploaded as separate, high-resolution files. The journal is now integrated with Wiley's Image Checking service. For further details, see: https://www.wiley.com/en-us/network/publishing/research-publishing/trending-stories/upholding-image-integrity-wileys-image-screening-service

You may also upload:

- 'Potential Cover Art' for consideration as the issue's cover image
- Appropriate Supporting Information (Video, audio or data set: see https://jp.msubmit.net/cgi-bin/main.plex?form_type=display_requirements#supp).

We look forward to receiving your revised submission.

If you have any queries, please reply to this email and we will be pleased to advise.

Yours sincerely,

Natalia Trayanova
Senior Editor
The Journal of Physiology

----------------

REQUIRED ITEMS

- Authors are required to fully disclose the use of any Generative AI (GAI) tools used in the preparation of their manuscript. This disclosure should also be included in the 'Methods' section (or a suitable location for article types not requiring Methods), and include the name(s), model and version of AI technology used, and exact details on the purpose for, and methods of, its use.

If no AI tool has been used, this should also be specifically stated within the manuscript.

----------------

EDITOR COMMENTS

Reviewing Editor:

The reviewers found that the authors have addressed most of the previous concerns. The remaining comments mainly concern the interpretation of some findings and the discussion of the relationship between HDF and IVPG metrics.

-----------------

REFEREE COMMENTS

Referee #1:

Authors have satisfactorily addressed my previous concerns and further improved the manuscript.

In my opinion the manuscript will be a useful scientific contribution when published.

Referee #2:

The revised manuscript is substantially improved compared with the original submission. The authors have addressed several important concerns, including clarification of the study objectives, improved physiological justification of the selected HDF metrics, implementation of mixed-effects statistical analysis, and more cautious interpretation of the findings. The manuscript is now clearer and more rigorous overall.

I have still some concerns that can be solved:

First, The manuscript now appropriately describes the work as "hypothesis-generating." However, several sections of the manuscript still interpret statistically significant metrics too strongly given the exploratory nature of the analysis. In particular, I encourage the authors to moderate statements such as:

- "better predict functional recovery after deTac than conventional echocardiographic indices" (Introduction),

- "capture the dominant mechanisms driving maladaptive remodeling" (Discussion),

- and "provide a powerful tool to distinguish responders from non-responders" (Discussion),

I would recommend that the authors:

- explicitly describe metrics such as the early-to-late systolic impulse ratio as exploratory waveform descriptors rather than mechanistically established biomarkers;

- avoid attributing specific physiological meaning (e.g., "force balance," "efficiency," or "flow redirection") unless directly validated;

- and clarify that the present study identifies candidate HDF features associated with remodeling, rather than demonstrating definitive mechanistic or predictive superiority over conventional indices.

The manuscript still does not fully clarify the mechanistic distinction between HDF and classical Doppler-derived intraventricular pressure gradients (IVPGs). While the revised text now discusses this relationship and acknowledges some limitations, it remains unclear what physiological information is uniquely captured by HDF beyond spatial or temporal integration of IVPG-related quantities.

Because several conclusions emphasize the added mechanistic or predictive value of HDF metrics, I recommend that the authors explicitly revise the Discussion and Conclusions to clarify the following points:

- HDF and IVPGs originate from closely related momentum-balance formulations and therefore should not be interpreted as fu ndamentally independent physiological entities.

- The present study does not directly compare HDF against Doppler-derived IVPG measurements.

- The current results therefore demonstrate feasibility and potentially complementary information, but not clear physiological or mechanistic superiority of HDF over established IVPG approaches.

- Some of the apparent robustness of HDF metrics may arise from spatial and temporal integration/averaging, and this possibility should be acknowledged explicitly.

---

END OF COMMENTS

EDITOR COMMENTS

REQUIRED ITEMS

- Authors are required to [<a]https://jp.msubmit.net/cgi-bin/main.plex?form_type=display_requirements#aigcfully disclose the use of any Generative AI (GAI) tools used in the preparation of their manuscript. This disclosure should also be included in the 'Methods' section (or a suitable location for article types not requiring Methods), and include the name(s), model and version of AI technology used, and exact details on the purpose for, and methods of, its use.

If no AI tool has been used, this should also be specifically stated within the manuscript.

To address this required item, the following has been added to the methods section on page 8 lines 16-18:

"The Google Gemini Pro 3 model was used to generate select R functions for data analysis and code was manually verified. This was the only use of Generative AI in the preparation of this manuscript."

----------------

EDITOR COMMENTS

Reviewing Editor:

The reviewers found that the authors have addressed most of the previous concerns. The remaining comments mainly concern the interpretation of some findings and the discussion of the relationship between HDF and IVPG metrics.

We thank you for the opportunity to make further revisions to this manuscript based on the reviewer's comments.

-----------------

REFEREE COMMENTS

Referee #1:

Authors have satisfactorily addressed my previous concerns and further improved the manuscript.

In my opinion the manuscript will be a useful scientific contribution when published.

We greatly appreciate the kind words and are pleased with the improvements to your manuscript based on your and the other reviewer's suggestions along with the recommendations of the reviewing editor.

Referee #2:

The revised manuscript is substantially improved compared with the original submission. The authors have addressed several important concerns, including clarification of the study objectives, improved physiological justification of the selected HDF metrics, implementation of mixed-effects statistical analysis, and more cautious interpretation of the findings. The manuscript is now clearer and more rigorous overall.

We are very appreciative of your supportive words and for the opportunity to continue to improve this manuscript.

I have still some concerns that can be solved:

First, The manuscript now appropriately describes the work as "hypothesis-generating." However, several sections of the manuscript still interpret statistically significant metrics too strongly given the exploratory nature of the analysis. In particular, I encourage the authors to moderate statements such as:

- "better predict functional recovery after deTac than conventional echocardiographic indices" (Introduction),
- "capture the dominant mechanisms driving maladaptive remodeling" (Discussion),
- and "provide a powerful tool to distinguish responders from non-responders" (Discussion),

We thank the reviewer for this comment. We agree it is important to stay consistent on language regarding predictive power of exploratory metrics. To address these concerns, we have made the following adjustments:

On page 5 lines 11-14, the following has been adjusted:
"Specifically, we tested whether interval-specific systolic HDF metrics capture changes in intraventricular force generation and flow turbulence induced by TAC, and whether these parameters may better predict functional recovery after deTAC than conventional echocardiographic indices."

On page 14 lines 7-10, the following has been adjusted:
"This suggests that, at least in the context of pressure overload and its relief, altered systolic force generation and turbulent flow within the ventricle capture ~~the dominant~~ mechanisms driving maladaptive remodeling, while diastolic HDF changes may be subtler, secondary phenomena."

On page 14 lines 14-16, the following has been adjusted:
Taken together, these results suggest that echocardiographic assessment of HDFs may provide a ~~powerful~~ tool to distinguish responders from non-responders after surgical treatment, thereby identifying individuals at risk of persistent hemodynamic dysfunction.

I would recommend that the authors:

- explicitly describe metrics such as the early-to-late systolic impulse ratio as exploratory waveform descriptors rather than mechanistically established biomarkers;

We thank the reviewer for this comment, and to address the concern we have updated the abstract on page 2 lines 12-15 to the following:

"Notably, a novel exploratory waveform, early-to-late systolic impulse longitudinal HDF ratio, showed the strongest associations with pressure overload and subsequent functional recover…"

- avoid attributing specific physiological meaning (e.g., "force balance," "efficiency," or "flow redirection") unless directly validated;

We thank the reviewer for this comment, to avoid specific physiological meaning where it is not specifically validated in our study, we have changed the abstract on page 2 line 2-4, the introduction on page 5 line 11-14, and the discussion on page 14 line 7-10 to the following respectively:

"Hemodynamic forces (HDF) quantify intraventricular pressure gradients as integrated vectors derived from myocardial motion and blood flow, providing a sensitive marker of left ventricular ~~pump efficiency~~ remodeling beyond conventional metrics such as ejection fraction or strain."

"Specifically, we tested whether interval-specific systolic HDF metrics capture changes in intraventricular force generation and flow ~~redirection~~ turbulence induced by TAC, and whether these parameters may better predict functional recovery after deTAC than conventional echocardiographic indices."

"This suggests that, at least in the context of pressure overload and its relief, altered systolic force generation and turbulent flow within the ventricle capture mechanisms driving maladaptive remodeling."

- and clarify that the present study identifies candidate HDF features associated with remodeling, rather than demonstrating definitive mechanistic or predictive superiority over conventional indices.

We thank the reviewer for this comment and address this concern with the previous response with our change to the abstract on page 2 line 2-3:

"Hemodynamic forces (HDF) quantify intraventricular pressure gradients as integrated vectors derived from myocardial motion and blood flow, providing a sensitive marker of left ventricular ~~pump efficiency~~ remodeling beyond conventional metrics such as ejection fraction or strain."

The manuscript still does not fully clarify the mechanistic distinction between HDF and classical Doppler-derived intraventricular pressure gradients (IVPGs). While the revised text now discusses this relationship and acknowledges some limitations, it remains unclear what physiological information is uniquely captured by HDF beyond spatial or temporal integration of IVPG-related quantities.

We thank the reviewer for this comment and have added in context through the responses to the reviewer's comments below regarding the discussion, limitations, and conclusions sections.

Because several conclusions emphasize the added mechanistic or predictive value of HDF metrics, I recommend that the authors explicitly revise the Discussion and Conclusions to clarify the following points:

- HDF and IVPGs originate from closely related momentum-balance formulations and therefore should not be interpreted as fundamentally independent physiological entities.

We appreciate this insight, and have made the following addition to the limitation section on page 14 line 30 and page 15 lines 1-2:

"Further, as HDF is a closely related momentum-balance calculation to IVPG, we acknowledge that it should not be interpreted as a fundamentally independent physiological metric."

- The present study does not directly compare HDF against Doppler-derived IVPG measurements.

We thank the reviewer for this suggestion and have made the following changes to the conclusion on page 15 lines 18-22:

"While future investigations incorporating 3D/4D HDF analysis, diastolic-focused assessments, direct comparison against doppler-derived IVPG measurements, and sex-balanced cohorts could refine their clinical translatability in pressure overload heart failure, this work demonstrates the potential of HDF waveform analysis to improve assessment of LV remodeling over standard echocardiographic measures."

- The current results therefore demonstrate feasibility and potentially complementary information, but not clear physiological or mechanistic superiority of HDF over established IVPG approaches.

We appreciate this comment and have made the following adjustments to the limitation section on page 14 lines 27-30:

"~~The~~ While the use of HDF metrics shows theoretical benefits over Euler IVPG, ~~but~~ we did not assess eccentric remodeling (Opie *et al.*, 2006) or a comparison against doppler-derived ~~so a direct benefit over~~ traditional Euler IVPGs, so a direct benefit is not explored in this study."

- Some of the apparent robustness of HDF metrics may arise from spatial and temporal integration/averaging, and this possibility should be acknowledged explicitly.

We thank the reviewer for this suggestion and have added the following to the limitations section to address this concern on page 14 lines 25-27:

"Despite variability, it is possible that the apparent robustness of HDF metrics could arise at least in part from the spatial and temporal averaging of 2D measurements used for integration."

## 2nd Revision - Editorial Decision 24-Jun-2026

Dear Dr Sicard,

Re: JP-RP-2026-290877R2 "**Ultrasound-Based Hemodynamic Force Trajectories in a Murine Model of Reversible Pressure Overload**" by Peyton Day, Thomas Moore-Morris, Craig J Goergen, and Pierre Sicard

We are pleased to tell you that your paper has been accepted for publication in The Journal of Physiology.

The last Word (or similar) version of the manuscript provided will be used by the Production Editor to prepare your proof. When this is ready you will receive an email containing a link to Wiley's Online Proofing System. The proof should be thoroughly checked and corrected as promptly as possible.

Authors should note that it is too late at this point to offer corrections prior to proofreading. Major corrections at proof stage, such as changes to figures, will be referred to the Editors for approval before they can be incorporated. Only minor changes, such as to style and consistency, should be made at proof stage. Changes that need to be made after proof stage will usually require a formal correction notice.

All queries at proof stage should be sent to: TJP@wiley.com.

Following copyediting and typesetting the accepted version will be published online.

Yours sincerely,

Natalia Trayanova
Senior Editor
The Journal of Physiology

IMPORTANT POINTS TO NOTE FOLLOWING ACCEPTANCE OF YOUR PAPER:

• IMPORTANT NOTICE ABOUT OPEN ACCESS: To assist authors whose funding agencies mandate immediate public access to published research findings, The Journal of Physiology allows authors to pay an Open Access (OA) fee to have their papers made freely available immediately on publication.

The Corresponding Author will receive an email from Wiley with details on how to register or log in to Wiley Authors where you will be able to place an order.

• You can check if your funder or institution has a Wiley Open Access Account here:
https://authors.wiley.com/author-resources/Journal-Authors/open-access/author-compliance-tool.html

MEMBER DISCOUNT: Corresponding authors who are Members of the Physiological Society are eligible for a 20% discount on the cost of the Article Processing Charge (APC). To claim this discount, the Corresponding Author will need to log in to the Physiological Society Membership portal and retrieve the discount code (https://www.physoc.org/journals-and-media/journals/journal-discount-for-members/). This code should then be quoted in the billing section of Wiley Authors when selecting the open access option.

• TRANSPARENT PEER REVIEW POLICY: To improve the transparency of its peer review process, The Journal of Physiology publishes online as supporting information the peer review history of all articles accepted for publication. Readers will have access to decision letters, including Editors' comments and referee reports, for each version of the manuscript as well as any author responses to peer review comments. Referees can decide whether or not they wish to be named on the peer review history document.

• You can help your research get the attention it deserves! Check out Wiley's free Promotion Guide for best-practice recommendations for promoting your work at: www.wileyauthors.com/eeo/guide. You can learn more about Wiley Editing Services which offers professional video, design, and writing services to create shareable video abstracts, infographics, conference posters, lay summaries, and research news stories for your research at: www.wileyauthors.com/eeo/promotion.

• If you would like to receive our 'Research Roundup', a monthly newsletter highlighting the cutting-edge research published in The Physiological Society's family of journals (The Journal of Physiology, Experimental Physiology, Physiological Reports, The Journal of Nutritional Physiology and The Journal of Precision Medicine: Health and Disease), please click this link, fill in your name and email address and select 'Research Roundup':
https://www.physoc.org/journals-and-media/membernews

----------------

EDITOR COMMENTS

Reviewing Editor:

I would like to congratulate the authors on their careful revisions and the quality of the final manuscript

-----------------

REFEREE COMMENTS

Referee #1:

Authors had satisfactorily addressed all my concerns.

Referee #2:

The authors have thoroughly addressed all of the concerns raised in the previous review round. The revisions have significantly improved the manuscript, and the responses provided are clear, complete, and satisfactory. I would like to congratulate the authors on their careful work and the quality of the revised manuscript.
